# Chlorophyll fluorescence as a tool for nutrient status identification in rapeseed plants

**DOI:** 10.1007/s11120-017-0467-7

**Published:** 2017-11-28

**Authors:** Hazem M. Kalaji, Wojciech Bąba, Krzysztof Gediga, Vasilij Goltsev, Izabela A. Samborska, Magdalena D. Cetner, Stella Dimitrova, Urszula Piszcz, Krzysztof Bielecki, Kamila Karmowska, Kolyo Dankov, Agnieszka Kompała-Bąba

**Affiliations:** 1 Institute of Technology and Life Sciences (ITP), Falenty, Al. Hrabska 3, 05-090 Raszyn, Poland; 2 White Hill Company, Żurawia 71/3, 15-540 Białystok, Poland; 3 Department of Plant Ecology, Institute of Botany, Jagiellonian University, Lubicz 46, 31-512 Kraków, Poland; 4 Department of Plant Nutrition, Wrocław University of Environmental and Life Sciences, Grunwaldzka 53, 50-357 Wrocław, Poland; 5 Department of Biophysics and Radiobiology, Faculty of Biology, St. Kliment Ohridski University of Sofia, 8 Dr Tzankov Blvd., 1164 Sofia, Bulgaria; 6 Department of Plant Physiology, Faculty of Agriculture and Biology, Warsaw University of Life Sciences – SGGW, Nowoursynowska 159, 02-776 Warszawa, Poland; 7 Department of Botany and Nature Protection, University of Silesia, Jagiellońska 28, 40-032 Katowice, Poland

**Keywords:** Nutrient-deficiency detection, Nutrient status, Chlorophyll *a* fluorescence, OJIP test, Machine learning, Super-organising maps

## Abstract

In natural conditions, plants growth and development depends on environmental conditions, including the availability of micro- and macroelements in the soil. Nutrient status should thus be examined not by establishing the effects of single nutrient deficiencies on the physiological state of the plant but by combinations of them. Differences in the nutrient content significantly affect the photochemical process of photosynthesis therefore playing a crucial role in plants growth and development. In this work, an attempt was made to find a connection between element content in (i) different soils, (ii) plant leaves, grown on these soils and (iii) changes in selected chlorophyll *a* fluorescence parameters, in order to find a method for early detection of plant stress resulting from the combination of nutrient status in natural conditions. To achieve this goal, a mathematical procedure was used which combines principal component analysis (a tool for the reduction of data complexity), hierarchical k-means (a classification method) and a machine-learning method—super-organising maps. Differences in the mineral content of soil and plant leaves resulted in functional changes in the photosynthetic machinery that can be measured by chlorophyll *a* fluorescent signals. Five groups of patterns in the chlorophyll fluorescent parameters were established: the ‘no deficiency’, Fe-specific deficiency, slight, moderate and strong deficiency. Unfavourable development in groups with nutrient deficiency of any kind was reflected by a strong increase in *F*_o_ and Δ*V*/Δ*t*_0_ and decline in *φ*_Po_, *φ*_Eo_ *δ*_Ro_ and *φ*_Ro_. The strong deficiency group showed the suboptimal development of the photosynthetic machinery, which affects both PSII and PSI. The nutrient-deficient groups also differed in antenna complex organisation. Thus, our work suggests that the chlorophyll fluorescent method combined with machine-learning methods can be highly informative and in some cases, it can replace much more expensive and time-consuming procedures such as chemometric analyses.

**Electronic supplementary material:**

The online version of this article (10.1007/s11120-017-0467-7) contains supplementary material, which is available to authorized users.

## Introduction

A doubling in global food demand over the next 50 years poses huge challenges for the sustainability of food production, terrestrial and aquatic ecosystems and the services they provide to society (Tilman et al. 2002). During the last century, the agricultural production steadily increased due to improved nutrient availability, and crop improvement and protection (Ludwig et al. 2011; Kalaji et al. 2014a). However, it often comes with environmental costs: water pollution, soil degradation, trace gas emission, climate changes and loss of biodiversity (Ludwig et al. 2011). Therefore, what is needed is the development of precise, quick and low-cost methods to evaluate nutrient content in plant tissue and soil which will allow producers to minimise the use of artificial fertilisers (Gomiero et al. 2011).

The 17 micro- and macroelements, including nitrogen (N), phosphorus (P), potassium (K), calcium (Ca), sulphur (S), magnesium (Mg), manganese (Mn), copper (Cu) and iron (Fe), are assumed to be essential to plant physiological functions and growth (Arnon and Stout 1939; Kabata-Pendias 2011). Additionally, their availability to plants strongly depends on several other factors, such as pH, hydrolytic acidity, soil granulometric composition and soil organic matter content. The relationship between the particular environmental resource and plant growth is described as a ‘generalised dose–response curve’ (Osman 2013; Kalaji et al. 2014a). However, in natural conditions, in contrast to experimental ones, single element deficiencies are rare. Rather, we are faced with the combinations of element demands and deficiencies. Thus, it is possible to find ranges of elements optimal for plant growth which could be related to the ecological niche concept (Hutchinson 1957; Chase and Leibold 2003).

Photosynthesis is the main process of plant metabolism, which is strongly influenced by environmental conditions (Kalaji et al. 2014b). This process consists of four phases (i) light absorption by the antenna system, (ii) primary electron transfer in reaction centres, (iii) energy stabilisation by secondary processes and (iv) the synthesis and transport of photosynthetic products (Blankenship 2014). The photochemical processes are driven by protein complexes: PSI, PSII, cytochrome *b*_*6*_/*f* and they occur in the thylakoid membranes of chloroplasts (Kalaji et al. 2014a). The incident light energy is absorbed by light-harvesting complexes of photosystems and transported to the central part of antenna (Rochaix 2011). As energy transfer takes place, the excitation energy moves towards the central parts of RC, from higher to lower energy pigments (Blankenship 2014). This results in charge separation across a membrane and the water being split into molecular oxygen protons and electrons on the donor side of PSII (Kalaji et al. 2014a). The electrons are transported from PSII to the plastoquinone pool (*Q*_A_, *Q*_B_) through *b*_*6*_/*f*, plastocyanin to PSI (Rochaix 2011). In PSI, the second charge separation occurs, which is followed by a reduction in ferredoxin. This finally reduces the NADP^+^ to NADPH. The electron transport is coupled with proton pumping, producing a pH gradient which drives the synthesis of ATP by ATP synthase (Rochaix 2011).

A deficiency in nutrients strongly influences the photosynthetic apparatus structure and functions, including PSII photochemistry (Kalaji et al. 2014a). It negatively affects the photosynthetic apparatus, mainly by the disrupted synthesis of key photosynthetic components. The directs effect on synthesis of specific protein complexes are confirmed for nitrogen, sulphur and iron deficiencies (Abadía 1992; Ciompi et al. 1996; D’Hooghe et al. 2013; Kalaji et al. 2014a; Jin et al. 2015). Chlorophyll synthesis is directly affected by a deficiency in nitrogen, magnesium and iron (Ciompi et al. 1996; Laing et al. 2000). Moreover, calcium and potassium play a crucial role in membrane stabilisation and cell signalling associated with the stress response (Qu et al. 2012). The photosynthesis is also affected by the P deficiency, mainly indirectly by slowing down ATP and NAPH production (Terry and Ulrich 1973); when plants suffer from P deficiency the parameters ϕ_PSII_, ETR and qP are decreased (Xu et al. 2007). Additionally, nutrient deficiencies lead to decreases in biomass and an imbalance in water relations.

In addition to biochemical and gas exchange methods, chlorophyll *a* fluorescence is assumed as reliable, a non-invasive technique for the assessment of electron transport and related photosynthetic processes (Kalaji et al. 2012, 2017; Bąba et al. 2016; Goltsev et al. 2016). For a quick assessment of the photosynthetic function in a high number of samples in the field conditions, a non-destructive analysis of fast chlorophyll transient was developed (Strasser et al. 1995, 2004). The method is based on the high-frequency recording of chlorophyll fluorescence (ChlF) emitted by dark pre-adapted leaf samples during the pulses of strong actinic light by a fluorometer. The fluorescence kinetics obtained in this way provide information on the structural and functional state of the photosystems, mainly PSII (Strasser et al. 2004; Stirbet and Govindjee 2011). The fluorescence curve rises from the minimal *F*_o_ to the maximal *F*_M_ value. The mathematical model, called the OJIP test, describes this polyphasic transient and enables the calculation of parameters, quantum yields and probabilities which give insight into PSII and PSI functions (Kalaji et al. 2014a).

Numerous studies have demonstrated the usefulness of the OJIP test in uncovering the differences in the chlorophyll fluorescence transient among plant genotypes, varieties or mutants (Brestic et al. 2012, 2015). Moreover, strong changes in ChlF transients have been observed in plants under different types of environmental stress (Kalaji et al. 2011, 2014b). There are examples of studies on the detection of nutrient deficiency in plants with the use of fast chlorophyll fluorescence (Lu et al. 2001; Kalaji et al. 2014a, 2016). The detail connection of the shape of a ChlF rise with the rate constants of electron transport reactions during the photosynthesis light phase has been analysed by comprehensive model descriptions of the processes that determine the efficiency of fluorescence emission by PSII antennae chlorophylls (Lazar 2003; Belyaeva et al. 2014). The ChlF transients can be treated as a “fingerprint” (Tyystjärvi et al. 1999)—formed by the parameters derived from OJIP transients or by comparing the modified shape of the fluorescence rise kinetics. This “kinetic response” often contains hidden information concerning the stress type, a specific plant tolerance to the applied stress and other important and interesting information related to the plant as a whole even though at first sight, it is not directly connected to the photosynthetic apparatus of the plant.

The identification of such kinds of hidden information is possible through additional secondary fluorescence data processing, using multivariate analyses such as the principal component analysis (PCA) or artificial intelligence methods which allow the analysis of large datasets, the amount, precision and complexity of which cannot be efficiently analysed by traditional methods (Samborska et al. 2014).

Using multivariate analyses similar to principal component analyses (PCA), it is possible to reduce the large set of Chl *a* fluorescence variables to the few most informative ones (Legendre and Legendre 2012; Goltsev et al. 2012) and in this way detect the main *trade-offs* among Chl *a* fluorescence parameters and remove errors from the dataset.

Machine-learning methods, such as artificial neural networks (ANNs) and self-organising maps (SOM), are powerful tools for Chl *a* fluorescence data analysis (Kohonen 2001; Kalaji et al. 2017). ANNs create artificial intelligence resembling the human brain (Kohonen 2001; Samborska et al. 2014; Kalaji et al. 2017) allowing big data analysis. SOMs are a special instance of neural network analysis in which apart from data mining the visualisation of complex multi-dimensional datasets is enabled on the two-dimensional plane. Both methods could be used for a *classification* of different responses of plants to various environmental factors: (i) *finding specific shapes* of Chl *a* fluorescence induction curves, (ii) determining the most important Chl *a* fluorescence parameters or points on the Kautsky curve that differentiate them. Moreover, we can also (iii) *predict the values* of other environmental or physiological variables on the basis of Chl *a* fluorescence data (Goltsev et al. 2012), Tyystjärvi et al. applied artificial intelligence methods to the analysis of fluorescence data induced by a sequence of different types of illumination (low light intensity, saturating pulse, far-red, etc.) in order to identify plant species (Tyystjärvi et al. 1999; Keränen et al. 2003; Codrea et al. 2003; Kirova et al. 2009).

In this study, chlorophyll fluorescence (ChlF), data reduction (PCA) and a novel machine-learning method—super-organising maps (sSOM) were used to develop a method or the non-invasive detection and monitoring of micro- and macronutrients deficiency in rapeseed grown in field conditions.

## Materials and methods

### Plant growth

Sixty soil samples representative of a wide range of arable soils coming from different parts of Lower Silesia, southwestern part of Poland were used as a substrate in the experiment. The pot experiment with rapeseed was carried out in a growth chamber. 500 g of soil (air-dried) was placed in pots in two replicates. The rapeseed (*Brassica napus* var. Monolit) plants were grown at 16–21 °C, 16/8 h photoperiod PPFD of 300 μmol m^−2^ s^−1^. The pot positions were randomised every week.

Soil samples and the leaves of rapeseed plants grown on these soils underwent a detailed analysis of their nutrient content by chemometric methods. The collected data were used as a reference for the results acquired from biophysical experimental methods and mathematical analysis. The entire experimental and data analysis procedures are illustrated in Supplemental Fig. 1.

### Soil and plant analysis

The bioavailable forms of nutrients in the soil were assessed with standard methods in Poland: magnesium (PN-R-04020:1994) by 0.0125 mol dm^−3^ CaCl_2_ originally by (Schachtschabel 1954), phosphorus and potassium (PN-R-04023:1996, PN-R-04022:1996) by adapted Double Lactate method 0.02 M Ca-lactate C_6_H_10_CaO_6_·5H_2_O + 0.02 M HCl, pH 3.6 (Egner et al. 1960), micronutrients in 1 mol dm^−3^ HCl method developed by Rinkis (1963) and applied to the Polish soil testing system by Gembarzewski et al. (1987), according to the Polish norm PN-R-04016:1992—zinc, PN-R-04017:1992—copper, PN-R-04019:1993—manganese. Moreover, soil organic carbon was assessed according to PN-ISO 14235:2003 and total nitrogen content by the Kjeldahl method.

Additionally, two other methods for soil testing were used: Mehlich 3 for simultaneous soil extraction of some bioavailable elements K, Mg, Ca, Fe, Mn, Zn, Cu (Mehlich 1984), and for the same element, the Yanai et al. (1999) method as these two tests are useful for acidic to near neutral soils.

Moreover, selected physical properties of the soil were measured: pH in 1 mol dm^−3^ KCl (PN-ISO 10390:1997), and particle size distribution of soils using the Casagrande method (PN-ISO 11277 2005).

The macro- and microelement contents of rapeseeds were analysed in order to find the relationship between the element content in plants tissue and the soil in which they were grown. The plant leaves were harvested on 25 and 40 DAS (days after sowing; BBCH-scale 14 and 15, respectively) and were mineralised at 450 °C in a muffle furnace, then, after ash dissolution in 6 mol HCl (Sillanpää 1982). They were analysed for nutrient content: Mg, Ca, Fe, Mn, Cu with atom absorption spectroscopy and K by atom emission spectroscopy. N and P were analysed by colorimetry using the indophenol method and Murphy and Riley Molybdenum Blue, respectively.

### Chlorophyll *a* fluorescence

The chlorophyll fluorescence (ChlF) measurements were performed at 25 and 40 DAS. The sampling was performed on the middle part of adaxial leaf blades away from the main leaf vein after additional dark adaptation (30 min) using leaf clips. Fluorescence measurements were performed with the Handy PEA fluorimeter (Hansatech Instruments, King’s Lynn, Norfolk, UK). Red actinic light (wavelength at peak 650 nm; spectral line half-width 22 nm) with the intensity of 3500 μmol m^−2^ s^−1^ was used for the induction of fluorescence and 1 s of transient fluorescence was recorded. The fluorescence signal was collected at a maximum frequency of 10^5^ points s^−1^ (each 10 μs) within 0–0.3 ms, after which the frequency of recording gradually decreased, collecting a total of 118 points within 1 s. ChlF transient data were used to calculate the basic parameters and the parameters needed for the OJIP test. The *F*_o_ level was measured as fluorescence at 20 μs. The collected data were used for the calculation of the basic parameters, while the fluorescence intensities determined at O-20 μs, J-2 ms, I-30 ms and maximum fluorescence, *P* ~ 300 ms (*F*_M_) were used for the calculation of the OJIP test parameters.

### Data analysis

Several step analysis intended to find the patterns in element contents in the soil and plant leaf tissue and relate them to the chlorophyll fluorescence parameters was used to identify nutrient deficiency in rapeseed individuals.

Principal component analysis (PCA) was used to identify the deficiencies of N, P, K, Ca, Mg and Cu, Fe Zn and their combinations in (i) soil samples used in the experiment and (ii) in the rapeseed leaf tissue (25 and 40 DAS). This method allowed a reduction of the variation of the large, multi-dimensional datasets to a few (usually 1–3) most informative axes, called principal components (PCs). Along these axes, samples were ordered according to increased or decreased element content. In the ordination plots, the PCA preserves the Euclidean distances among samples, which means that closer samples are similar in terms of element content, while those which lie on the opposite sides of the axes are most dissimilar to each other (Legendre and Legendre 2012). PCA enables the variables (in this case the particular element content) to be found which are highly correlated with these PCs and therefore of the highest importance in explaining the differences observed in the data.

In the next step, the groups of (i) soil types, and (ii) leaves (25 and 40 DAS) with different micro- and macroelements content were obtained. Hierarchical k-means (h-k-means) classifications designed to find the regions of high-density of points in the space defined by the PCA axes were performed (Reddy et al. 2016). The h-k-means iteratively minimise the within-groups sum of squares until the optimal number of clusters is reached (Borcard et al. 2011). The results are presented on the PCA diagrams with superimposed h-k-means classifications. The dendrograms based on minimum variance (Ward’s method) and Euclidean distance (marked ‘height’ on the charts) were also constructed for better visualisation taking into account the hierarchical nature of relationships among the element content of the soil and leaf tissue samples.

The significance of the differences in the soil and plant leaves in the resulting groups was tested by one-way-ANOVA with a subsequent Tukey honest difference test. Inspection of group means for each element allowed the separation of the group(s) with significantly higher and lower values. The first one was treated as a ‘control’ to which samples with particular element deficiency were compared.

The groups defined on the basis of leaf element contents after 25 DAS were used in the classifications of ChlF parameters. These groups were chosen because (i) they show the clear ChlF parameter pattern for most of the elements studied, and (ii) the nutrient stress detected at the early stage of growth allowed for the application of deficient element(s), before stress irreversibly influences plant growth.

Super-organising maps were used to summarise the relationship between the pattern and values of ChlF parameters and pattern and values of leaf element contents.

Self-organising maps, in their base form, are a powerful unsupervised exploratory analysis, which can be thought of as a spatially constrained form of k-means clustering (Wehrens and Buydens 2007). This method has an advantage over PCA, as it can present more than two dimensions into the plane. SOM reproduce topology among objects instead of Euclidean distances (Kohonen 2001). Thus, if high-dimensional objects are similar, their position on the SOM-plane is close to each other. Moreover, rather than mapping objects on the continuous space, SOM use a grid of ‘neurons’ onto which the objects are mapped (Kohonen 2001; Samborska et al. 2014; Kalaji et al. 2017).

Super-organising maps (sSOM) is an extension of SOM, since it accounts for individual data types by using a separate layer for each data type (Wehrens and Buydens 2007). Leaf element content after 25 DAS (N, P, K, Ca, Mg, Cu, Zn, Fe and Mn), previously selected ChlF parameters (*F*_o_, Δ*V*/Δ*t*_0_, PI_Total,_ *δ*_Ro,_ *φ*_Po_, *φ*_Ro,_ *φ*_Eo_ and *γ*_RC_, see “Results”) and previously used h-k-means classification based on 25 DAS leaf element content were used as inputs for the model.

All analyses and calculations were performed with R CRAN version 3.3.1 (R Core Team 2016). The analysis of variance with a Tukey post-hoc test was performed with R core package *stats*. PCA analysis with ordination diagrams, h-k-means clustering analysis and dendrograms were obtained with the use of the R *vegan* 2.4-4 package (Oksanen et al. 2016), while the sSOM analysis and diagram were generated by the R *kohonen* 3.0.2 package (Wehrens and Buydens 2007).

A detailed explanation of the meaning of each ChlF parameter used is presented in Parameters description (Supplemental Table S6).

## Results

### Micro- and macroelement content in the soils

**Fig. 1:**
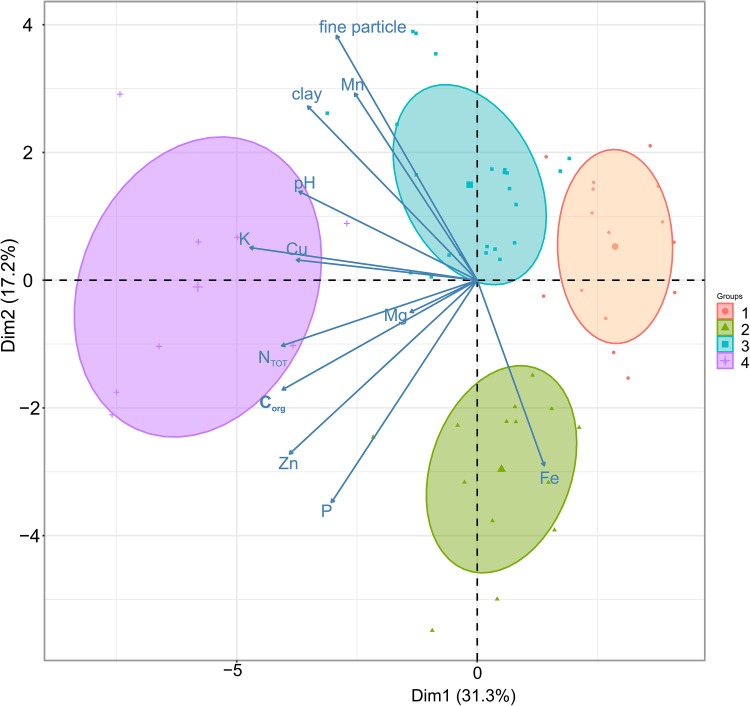
Principal component analysis (PCA) of 60 soil samples in terms of selected physical–chemical properties coming from different parts of the Lower Silesia, southwestern part of Poland. These soils were used in the experiments as a substrate for rapeseed plants (*Brassica napus* L. var. *napus*). This method allowed a reduction in the variation of the large, multi-dimensional datasets to a few most informative axes called principal components (PCs). The PCA preserves the Euclidean distances among samples, which means that closer samples are similar in terms of element content while those which lie on the opposite sides of the axes are most dissimilar to each other (Legendre and Legendre 2012). It enables also the finding of the variables (in this case the particular element content) highly correlated with these PCs. Two first axes which explained 31.1 and 17.2% variation in the data were presented. The four classes (marked with different colours) which resulted from the hierarchical k-means classification algorithm were superimposed onto the graph. On all PCA diagrams, the gradients of element concentrations were shown with arrows whose length and angle on the PCA axes are proportional to the strength of the correlation with these PCs. The direction of the arrows points to the increase in the content of this element, while the opposite direction points to their deficiency

60 different agricultural soil samples were tested representing the wide range of the variation in nutrient content. The PCA analysis with hierarchical k-means clustering revealed the main gradients in the physico-chemical compositions of soils. It enabled four optimal groups of samples to be distinguished, representing the presence or deficiency of the investigated elements. The detailed hierarchical classification of the soils, based on Ward’s method and Euclidean distance (denoted as ‘height’ in the dendrograms), also confirmed the results of the previous classification (Fig. 1, Supplemental Fig. S2).

On all PCA diagrams, the gradients of element concentrations were shown with arrows, whose length and angle on the PCA axes are proportional to the strength of the correlation with these PCs. The direction of the arrows points to the increase in the content of this element, while the opposite direction points to their deficiency. The first PCA axis, which explains the 31.3% variance in the soil data, separates Groups 1 and 4, the first one with lower average values of pH (5.37 vs. 6.76) and content of particle fraction (< 0.02 mm). Moreover, Group 1 appears to be highly deficient in the most important elements such as K, Cu, N_TOT,_ Zn, P, Mg, Fe and in C_org,_ in comparison to Group 4, which retains suitable concentrations of all needed nutrients (Fig. 1, Supplemental Fig. S2, Table S1).

The second PCA axis, which explains the 17.2% variance, separates the highly Zn and P-deficient Group 3, from Group 2 with lower Mn content (Fig. 1, Supplemental Table S1).

### Leaf element content

**Fig. 2:**
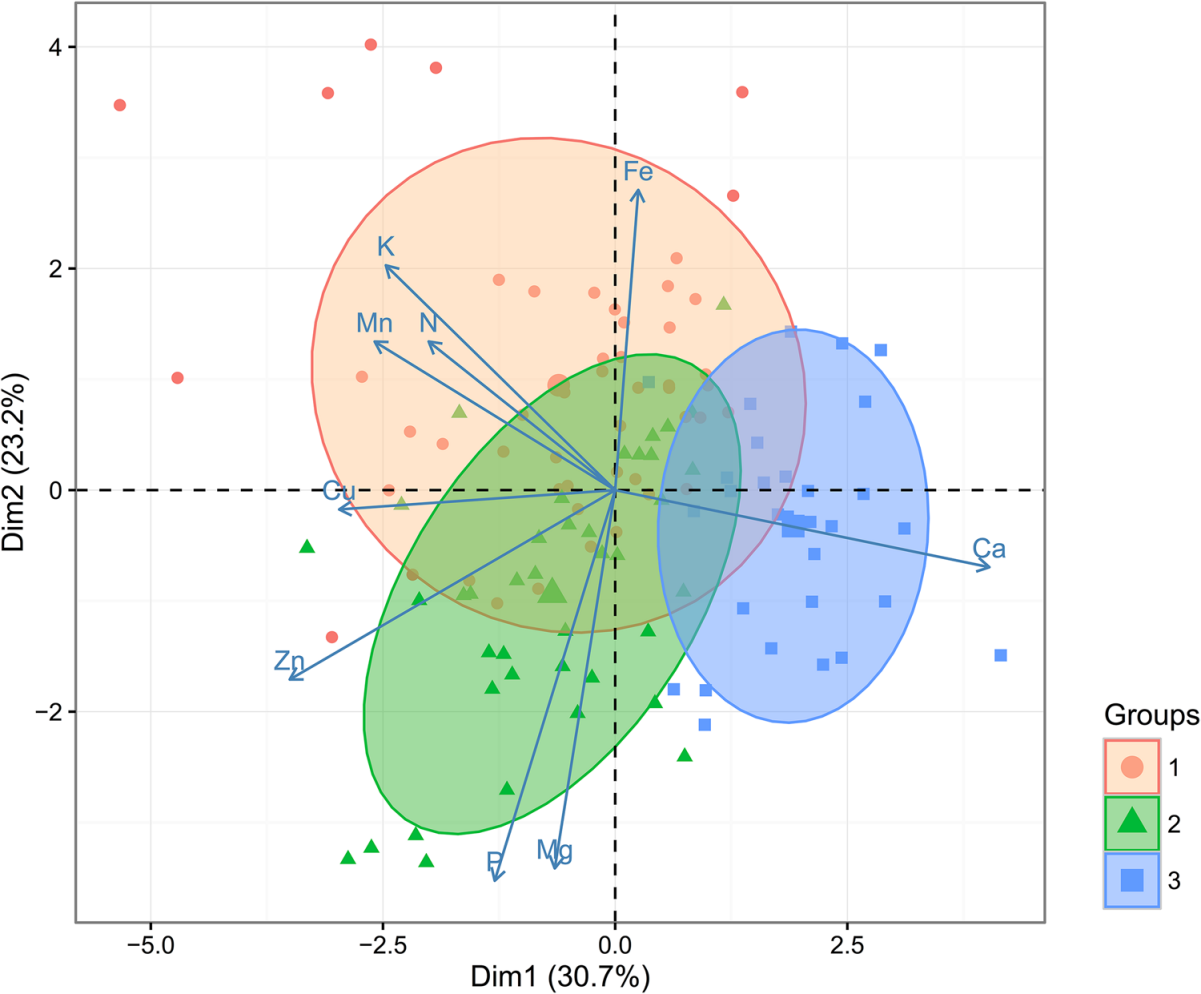
Results of the principal component analysis of leaf micro- and macroelement content in rapeseed leaves 25 days after sowing (25 DAS). The three optimal classes (marked with different colours) which resulted from the hierarchical k-means classification algorithm were superimposed onto the graph

The pattern of differences in the soil data was reflected in the leaf nutrient content of plants analysed on 25 DAS. The PCA with hierarchical k-means clustering analysis revealed the main gradients in the plant leaf chemical compositions and the optimal three groups of samples related to different leaf element contents. The leaf samples from all three groups significantly differed in N and K content. The first axis, which explains the 30.7% variance in the dataset, separates the leaf samples from Groups 1 and 2 with higher Cu and Zn from Group 3, which seems to be deficient in all micro and macronutrients in spite of Ca. On the other hand, the second axis, which explained the 23.2% variation, separates the Group 2 of leaf samples with higher Mg and P contents and Group 1 with higher Fe (Fig. 2, Supplemental Figs. S3–S6, Table S2). However, the 95% confidence ellipses around Group 1 and Group 2 overlapped, thus they share some of the similarities in their nutrient content.

The hierarchical classification analysis (dendrogram on Supplemental Fig. S3) confirmed the presence of 3 groups when the dissimilarity parameter threshold (‘height’) for the partitioning of leaf samples is set to 0.6. In order to receive a more detailed picture, the value of this parameter was changed to 0.5, and 4 different groups were formed (Supplemental Fig. S3). A new PCA diagram with 4 groups according to nutrient content was obtained with the same correction (Supplemental Figs. S4, S6).

The new h-k-means classification based on PCA analysis confirmed the previously observed pattern. However, a new Cluster 4 appeared, which contained the Zn-deficient leaf samples, and Cluster 3 with N, K and Mn deficiencies (Supplemental Fig. S4).

The PCA on the element concentration in the leaf tissue of older plants (40 DAS) presented four clearly divided Groups: 1–3 with significantly higher leaf N, Group 1 with higher K and Mn and groups 3 and 4 with higher Ca content (Supplemental Fig. S7, Table S2). Group 2 constituted all necessary nutrient compounds except K, Group 3 had a high amount of Ca and Fe, but experienced deficiency in the rest of the investigated elements. The area of overlapping was much smaller than the area observed in plants, measured on 25 DAS, and the PCA diagram is very similar to the diagram of the nutrient content of the 60 soils (Fig. 1). This result suggests that after longer periods of time plants grown in a specific soil have utilised most of the existing available macro- and micronutrients and experience the lack of elements to a greater extent than after 25 DAS. Supplemental Fig. S8 provides more details.

### Selection of ChlF parameters and sSOM analysis

The differences in leaf nutrient content after 25 DAS were clearly reflected by changes in ChlF parameters. In nutrient-deficient Group 3, the significantly lower values of ChlF parameters *F*_o_, Δ*V*/Δ*t*_0_, *φ*_Po_, *φ*_Eo,_ *δ*_Ro_, *φ*_Ro_ and higher *F*_V_, *F*_M_, N, *V*_j_, *V*_i_, ABS/RC, DI/RC as compared to other groups were recorded. Moreover, changes in the specific energy fluxes, quantum yields of PSII but not performance indices (PI_ABS_) between three groups were noted (Supplemental Fig. S9, Table S3). The classification of this dataset into 4 groups provided more detailed information and was used in the sSOM analysis (Supplemental Table S4). The changes in values of most ChlF parameters in all 4 groups after 40 DAS confirmed the increased stress level as a result of increased nutrient deficiency (Supplemental Table S5). These analyses enabled the selection of 8 ChlF parameters and described different aspects of the functioning of photosynthetic apparatus, which were used in the sSOM model.

**Fig. 3:**
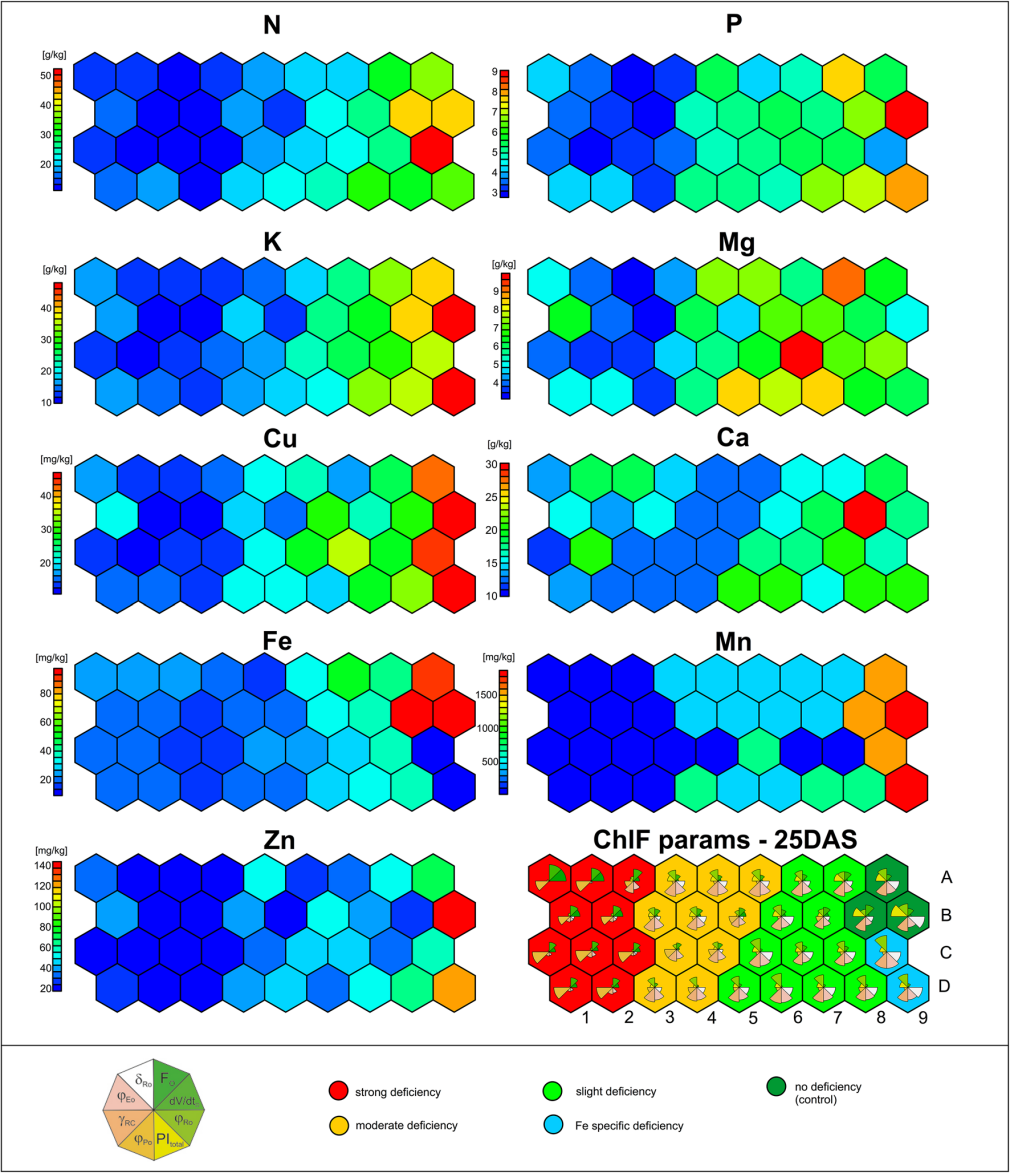
Relationship between leaf nutrient element content (LEC) after 25 days after sowing (25 DAS) and selected chlorophyll fluorescence parameters (ChlF) analysed by sSOM. This analysis accounts for individual data types (LEC and ChlF) by using separate layers. On the sSOM charts, the circles (36) are related to particular neurons. On the LMC layers, different colours are related to average values of particular leaf element content. On the ChlF layer, the values of *F*_o_, d*V*/d*t*_0_, PI_tot_ and *φ*_Ro_ are presented on the pie charts inside sSOM neurons. Moreover, classification of ChlF patterns into the five classes (marked with different background colours) based on the hierarchical k-means classification algorithm was superimposed onto this graph

The sSOM analysis of leaves after 25 DAS, enabled deeper investigation of the relationships between the ChlF and leaf element content and resulted in changes in ChlF parameters in response to particular element deficiency or combinations of them. In Fig. 3, each of the 10 charts represent the same sSOM topology: 36 neurons (hexagons) arranged into a 9 × 4 grid. Nine of them present the distribution map of leaf element content (LEC), while the average ChlF parameters are presented in pie charts on the 10th (bottom-right). The biggest size of classes inside the pie charts indicates the highest value of that element content.

In the LEC charts (Fig. 3), the different colours inside the hexagons are related to the averages of the particular leaf element content for the neurons and their values are shown on side bars. The sSOM analysis revealed a strong gradient visible from the left to the right side of the chart.

Leaf samples which were classified to neurons located on the rightmost side of the charts are nutrient-rich, while those on the leftmost side appear to be nutrient-deficient. The sSOM revealed five groups based on patterns on ChlF parameters and different LEC contents. They are marked with different background colours on the bottom-right panel of Fig. 3 The ‘dark-green’ group represents the all nutrient-rich leaves and is characterised by highest values of ChlF parameters: *φ*_Po,_ PI_Total_ and *φ*_Eo_. This group could be treated as a control (‘no deficiency’) and enabled the optimal doses of mineral nutrients for rapeseed to be specified (Table 1). The second, ‘light green’ group consists of individuals with significantly, but only slightly lower levels of leaf element content (‘slight deficiency’ group in Fig. 3; Table 1). This level of deficiency is related to a sharp decrease in PI_Total_, but did not influence *φ*_Po_. The third, ‘moderate deficiency’ (presented in orange) group, consists of individuals whose leaves contained significantly, but slightly lower element content both in comparison to the control as well as the ‘slight deficiency’ group. This group showed an increase in Δ*V*/Δ*t*_0_ and decrease in *φ*_Po,_ PI_Total_ and *φ*_Eo_. The fourth, ‘strong deficiency’ (red on Fig. 3) group of leaf samples deprived from all nutrients showed a strong increase in *F*_o_ and Δ*V*/Δ*t*_0_. In addition to the previously distinguished four groups, another ‘Fe-specific deficiency’ group was found (shown in blue). It is related to the specific Fe deficiency and showed a strong decrease in PI_Total_ in comparison to the control.

**Table 1:** Comparison of values of selected measured and calculated chlorophyll *a* fluorescence parameters (ChlF) and average element contents in plant leaves 25 days after sowing in rapeseed plants in 5 groups resulting from the super-SOM analysis

	No deficiency	Fe-specific deficiency	Slight deficiency	Moderate deficiency	Strong deficiency
Leaf nutrient content after 25DAS					
N (g kg^−1^)	45.92 ± 3.66a	32.90 ± 1.74b	24.76 ± 5.55c	17.53 ± 4.34d	13.38 ± 3.15e
P (g kg^−1^)	6.41 ± 1.54a	7.13 ± 0.86a	5.30 ± 1.40b	4.50 ± 1.16c	3.40 ± 0.77d
K (g kg^−1^)	42.23 ± 5.28a	43.28 ± 4.95a	25.66 ± 7.37b	16.41 ± 5.18c	12.80 ± 3.64d
Ca (g kg^−1^)	19.67 ± 4.78a	18.64 ± 2.29ab	17.34 ± 2.74b	12.42 ± 2.09c	14.70 ± 3.66d
Mg (g kg^−1^)	6.39 ± 1.26a	5.78 ± 0.30b	7.45 ± 1.55a	5.86 ± 1.95b	4.07 ± 1.19c
Cu (mg kg^−1^)	44.53 ± 7.03a	42.25 ± 3.33a	35.00 ± 6.71b	19.16 ± 5.91c	13.89 ± 3.09d
Fe (mg kg^−1^)	82.65 ± 17.00a	51.8 ± 44.01b	35.50 ± 8.90c	20.85 ± 5.65d	19.66 ± 7.45d
Mn (mg kg^−1^)	913.55 ± 37.42a	1216.25 ± 234.82b	38.84 ± 4.98c	31.63 ± 7.30c	17.33 ± 11.29c
Zn (mg kg^−1^)	93.32 ± 46.63a	95.8 ± 29.70a	39.23 ± 14.15b	31.90 ± 18.75c	20.62 ± 7.14d
Chlorophyll fluorescence parameters after 25DAS					
*F*_o_	809.70 ± 382.79a	500.00 ± 156.74b	734.63 ± 121.97c	950.06 ± 152.38ac	1948.00 ± 386.35d
Δ*V*/Δ*t*_0_	1.13 ± 0.08a	0.86 ± 0.23b	1.09 ± 0.07a	1.27 ± 0.07c	1.42 ± 0.20d
*φ*_Po_	0.79 ± 0.04a	0.77 ± 0.07b	0.76 ± 0.04ab	0.72 ± 0.05c	0.39 ± 0.14d
*φ*_Eo_	0.37 ± 0.11a	0.40 ± 0.09b	0.36 ± 0.05a	0.32 ± 0.04c	0.14 ± 0.06d
*δ*_Ro_	0.35 ± 0.04a	0.36 ± 0.04a	0.39 ± 0.03b	0.21 ± 0.07c	0.19 ± 0.04d
*φ*_Ro_	0.13 ± 0.04a	0.15 ± 0.04a	0.14 ± 0.02a	0.07 ± 0.03b	0.03 ± 0.01c
PI_Total_	7.82 ± 1.26a	4.48 ± 1.20b	6.15 ± 1.36c	2.73 ± 1.32d	1.64 ± 0.66e
ABS/RC	0.45 ± 0.07a	0.36 ± 0.06b	0.40 ± 0.01c	0.32 ± 0.05b	0.17 ± 0.07d
*γ*_RC_	0.74 ± 0.04a	0.69 ± 0.03ab	0.71 ± 0.01b	0.76 ± 0.03c	0.85 ± 0.05d
RC/Cso	271.53 ± 46.27a	217.88 ± 32.12b	294.46 ± 47.55bc	303.33 ± 47.48c	319.58 ± 74.49d

The means ± SE for four groups were presented. The values with the same letters were not significantly different at *p* < 0.05. according to Tukey honest difference test

**Fig. 4:**
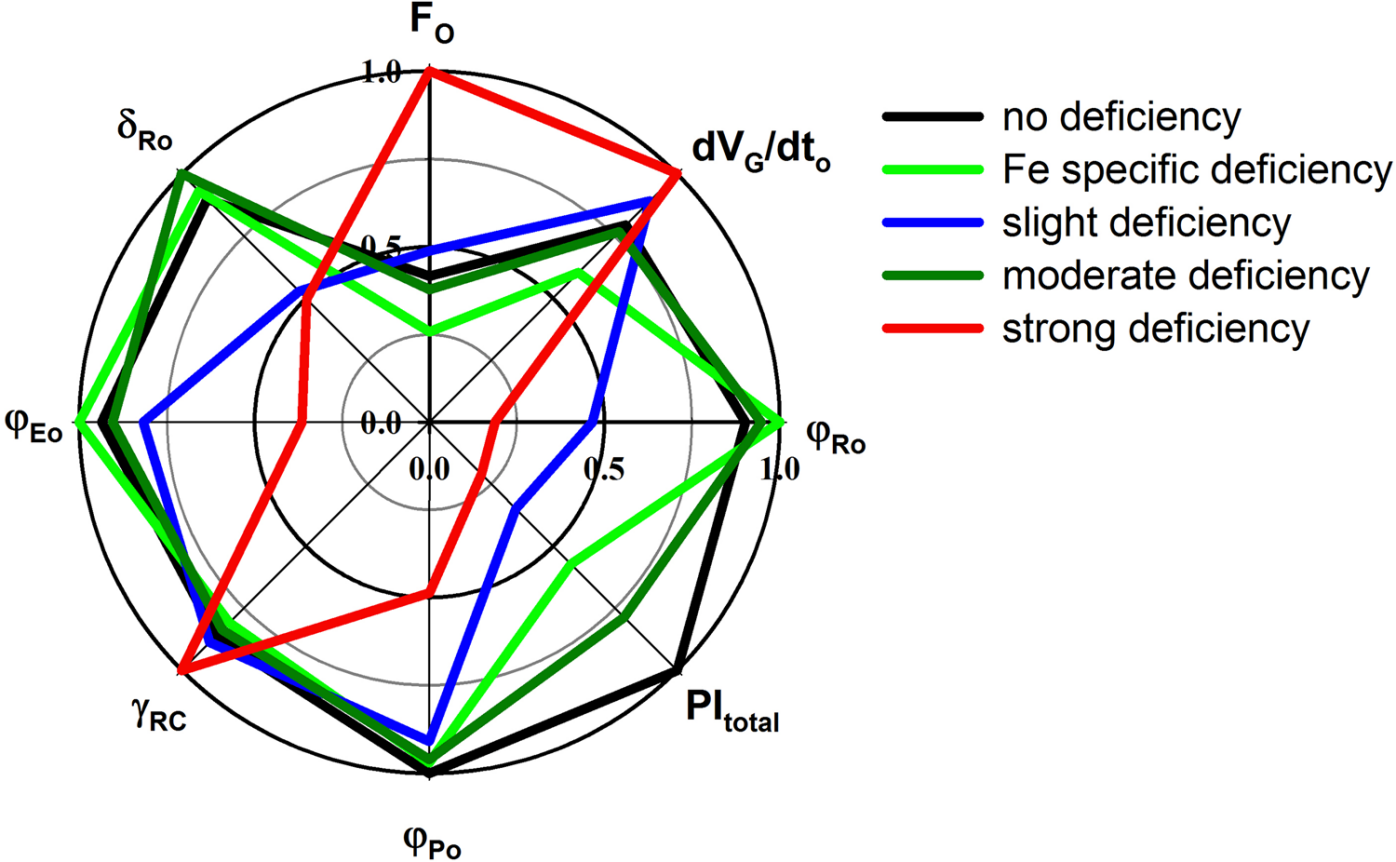
Comparison of JIP parameters for selected micro- and macronutrient deficiency. All the element values were normalised (divided by the maximal value) to enable the comparison of the variables measured on different scales

Analyses of the changes in the photosynthetic parameters calculated from the chlorophyll fluorescence induction curves for groups used in sSOM were performed by a method developed by Reto Strasser, called the OJIP test (Strasser et al. 2004). The OJIP test of 8 parameters showed clear patterns of the varying nutrient content impacts on the photosynthetic characteristics of rapeseeds (Fig. 4). Plants belonging to the sSOM groups ‘no deficiency’, ‘moderate deficiency’ and ‘Fe-specific deficiency’ have similar photosynthetic characteristics although they differ in the value of PI_Total_, which is one of the most sensitive OJIP parameters. Fe-specific and moderate deficiency groups contain plants with lower PI_Total_ due to lower overall productivity in comparison to the plants grown in soil, belonging to the ‘no deficiency’ group. The plants from the ‘slight deficiency’ group experience very different photosynthetic patterns—the lower *δ*_Ro_ and *φ*_Ro_ indicate improper electron transfer between the two photosystems and towards the end electron acceptors of PSI. This is also reflected in the lower level of the PI_Total_ parameter for these plants. Plants belonging to the group ‘strong deficiency’ exhibit the most varying photosynthetic profiles. Higher *F*_o_ corresponds to less efficient energy transfer among the antennae complexes toward the PSII reaction centre. The lower quantum yield *φ*_Po_ is a sign of strong inhibition. This combined with the lower *δ*_Ro_, *φ*_Ro_ and *φ*_Eo_ shows that the suboptimal development of the photosynthetic machinery affects both PSII and PSI. This is also confirmed by the low PI_Total_, observed in this group.

The chlorophyll *a* fluorescence curves of the established groups are presented in Supplemental Fig. 10. The shape of the induction curves varies greatly. Rapeseeds that experience deficiency of any kind exhibit higher levels of *F*_o_, which is typically observed in plants with damaged antennae complexes that cannot transfer energy efficiently to the reaction centre. Rapeseeds grown in less favourable conditions have developed fine-connected antennae complexes. Moreover, the higher level of *F*_M_ in these plants probably relates to less efficient electron transport between the acceptors of PSII and PSI, which leads to a full reduction of the reaction centres and higher quantum yield. Rapeseeds deficient in all nutrients show completely abnormal development of their photosynthetic machinery.

**Fig. 5:**
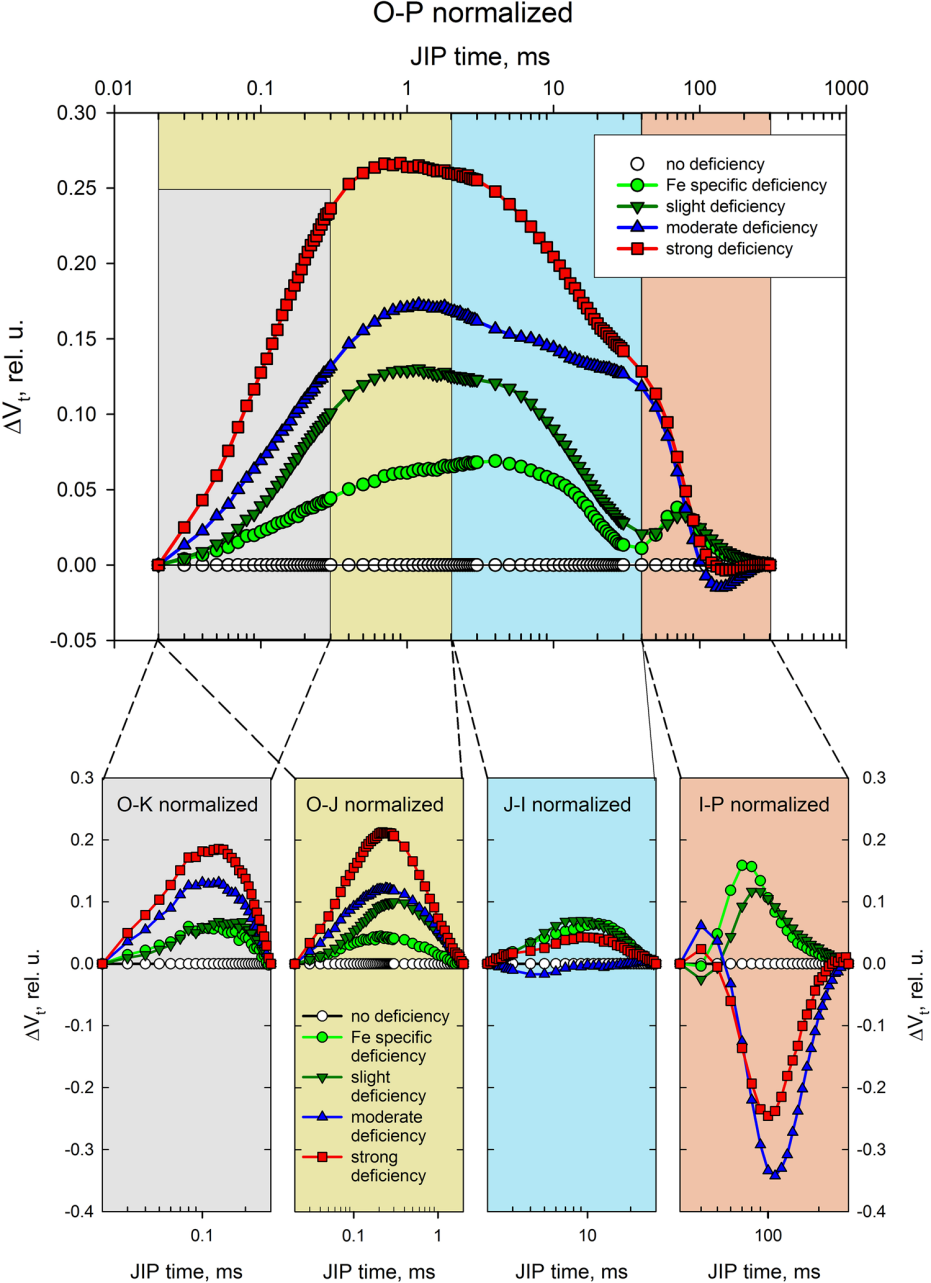
Differential chlorophyll fluorescence curves normalised between O–K, O–J, J–I and I–P

Different parts of this polyphase induction curve provide information on different steps in the photosynthetic process. Analysis of specific sections can in some cases provide more detailed information which is harder to process from analyses of the entire curve. Figure 5 presents differential curves obtained from the relative variable chlorophyll fluorescence transients taken at a specific time interval after the beginning of recording and then subtracted from the control sample transient (which in this case was calculated by means of double normalisation from the induction curve, measured in plants belonging to the group containing all necessary elements).

The O–K normalised curves called L bands provide information on the connectivity between RCs and their antennae complexes (Tsimilli-Michael and Strasser 2013a), which is important for the efficient light absorption and utilisation of the absorbed energy in the initial step of the photosynthetic process. A positive O–K band is a sign of less-connected antennae complexes which can be due to improper membrane organisation and lead to lower energy transfer and absorption efficiency. All groups with some specific nutrient deficiency show positive O–K bands and the group experiencing the least amount of micro- and macroelements presents the highest band. Another effect is the band peak shift to the longer time periods, which can be due to slower energy transfer to the RCs. Differential curves calculated between *F*_o_ and *F*_J_ are used to examine the condition of the donor side of PSII (Tsimilli-Michael and Strasser 2013b). Positive O–J normalised bands indicate an imbalance between the donation of electrons from the oxygen-evolving complex (OEC) to the oxidised PSII reaction centre chlorophyll (P680^+^) and the re-oxidation of reduced PSII acceptors (Q_A_^−^). This is usually due to impaired OEC, which is very sensitive to suboptimal conditions (Strasser 1997). J–I normalised and I–P normalised curves demonstrate an imbalance between the reduction and oxidation of Q_A_ and the plastoquinone pool, respectively (Tsimilli-Michael and Strasser 2013b). The latter depends on the efficiency of the electron uptake from the PSI acceptors and the number of available oxidised forms of NADP. Groups with moderate and strong deficiencies exhibit a negative band, which corresponds to a greater number of NADP+ molecules per active RC. This might be a compensatory mechanism developed by these plants in response to the suboptimal nutrient environment they were grown in.

## Discussion

Providing the best resources and the most suitable conditions is essential to plant growth and development and one of the main goals of modern agriculture (Osman 2013; Kalaji et al. 2014a). Climate changes and natural resources depletion along with the increasing need for long-term food safety require more comprehensive studies of plants physiology and their individual reaction to different environmental conditions (Tilman et al. 2002).

Different species vary greatly in their temperature optimum, illumination preferences, water uptake, micro and macronutrient needs and utilisation of the soil compounds, related to the ‘ecological niche’ concept (Chase and Leibold 2003). In order to provide them with the environment they need for growth and sustainable development, it is important to analyse each plants’ personal nutrient utilisation profile in detail, which is the goal of modern ‘precision agriculture’ (Bongiovanni and Lowenberg-Deboer 2004).

Plants tolerance and physiological state can be evaluated with various approaches in the fields of biochemistry, ecology, plant physiology, genetics and biophysics. In this study, measuring the chlorophyll *a* fluorescence was proposed as the most suitable of all methods, because it allows the gathering of huge amounts of detailed data about each step of the photosynthetic process, which is very sensitive to changes in a plant’s in vivo physiological state (Strasser et al. 2004; Kalaji et al. 2014a, 2017; Goltsev et al. 2016).

Deficiency in nutrients disrupts the functioning of the photosynthetic apparatus (Smethurst et al. 2005). The OJIP test is frequently applied in studies of nutrient deficiency essential to photosynthesis such as N, P, Cu, Fe, Mn (Larbi et al. 2004; Redillas et al. 2011; Tang et al. 2012; Schmidt et al. 2013; Kalaji et al. 2014a, 2016; Jin et al. 2015). However, in an experimental approach, the effects of single nutrient deficiency were studied by comparison of single element deficient samples with controls (plants fully saturated with elements crucial to plant life). Under natural conditions, however, micro- and macronutrients are present in different concentrations and forms, and in varying availability to the plants. This causes the necessity of the application of data mining methods in order to discover and classify a variety of patterns of content micro- and macronutrients in soil and in plant tissue (leaves), and to find the relationship between them and patterns of chlorophyll fluorescence parameters. This plant’s profiling can be achieved only by processing huge amounts of experimental data which are best executed with reliable numerical and statistical methods such as PCA and SOM analyses (Samborska et al. 2014; Kalaji et al. 2017).

This study used the explicit approach to understand a plant’s uptake of nutrients according to the constitution of soils in which the external factors such as temperature, soil moisture, illumination intensity, day/night period and position of pots in relation to the light source were experimentally controlled, and the effect of diversification of various soils with different nutrient contents is observed. Moreover, it was very important to draw a clear connection between the nutrient concentrations estimated in the soil, leaves and the values of relative difference of the fluorescent parameters. Inspection of Supplementary Fig. S11A–F confirmed a clear correlation between element contents in the soil, especially N, Mg, Cu, Mn, Zn and Fe in the soil and plant leaves after 25 DAS. The relationship is best visible for soils analysed by the Mehlich 3 method and usually on soils with pH below 5.5.

The sSOM method used in this study is a novel machine-learning method that can utilise huge amounts of high-dimensional data to create several two-dimensional visualisations (layers). On each layer, similar objects lie close to each other and could be presented in a form of ‘maps’, which are arranged according to common features. This allowed for the direct comparison of the patterns of nine investigated leaf element contents after 25 DAS, the corresponding values of 8 ChlF parameters and classification of leaves previously obtained from PCA and h-k-means. Each layer is characterised with 9 colour diagrams, which present the relative concentration of the element in the leaves (Fig. 3). For example, neuron 9D fits in a sSOM-arranged group, described as deficient in Fe which corresponds to the low concentrations of Fe observed in this position in the Fe diagram (concentration is below 20 mg/kg, which is considered far from the optimum for rapeseeds). Plants seeded on Fe-deficient soils exhibit low PI_Total_ and *γ*_RC_ which can be due to the formation of inactive reaction centres in PSII. This enabled a very detailed analysis to be performed for each neuron (Fig. 3).

Plants grown in 60 different soils with varying nutrient contents have developed typical changes in their photosynthetic machinery tuning their properties to the specific environment in order to achieve the highest possible productivity that can be reached in these conditions. This leads to the formation of groups of plants that express common photosynthetic characteristics in response to nutrient-deficiency stress. In this case, ‘stress’ was considered as any condition different from the optimum for the plant species (Fig. 3). The plants were not subjected to the crucial effect of a specific stress such as the total lack of a given important nutrient, but were initially grown in conditions with varying concentrations of elements which happened to be relatively deficient of different nutrients. The way these plants developed under varying conditions was studied and the observed effects were not exactly caused by adaptation to a stressful situation, but were a result of the plants abilities to develop different qualities according to the availability of nutrients in the soil. The chlorophyll *a* fluorescence parameters were sensitive enough to reflect the differences in the photosynthetic process.

A sSOM analysis based on chlorophyll fluorescence parameters created five groups according to the relative values of the eight chosen fluorescent parameters in the plants studied: no deficiency—which is fulfilled with optimal (or excess) concentrations of all elements and is not particularly deficient in any of them—slight deficiency, moderate deficiency, strong deficiency groups and an Fe-specific deficiency group, presented mainly by two neurons (9C and 9D).

These sSOM groups can also be analysed in more detail by examination of the induction curves, differential curves and the OJIP test. The effect of nutrient deficiency on the photosynthetic machinery of rapeseed caused by the lack of different elements is studied by measuring the chlorophyll *a* fluorescence emitted by leaves after illumination with photosynthetic active radiation (PAR). When a dark-adapted, green leaf is illuminated with PAR, a part of the energy absorbed by the antennae complexes of Photosystem II is emitted as fluorescence. The intensity of the fluorescent radiation depends on the efficiency of other relaxation processes such as photochemistry, energy transfer between complexes and heat dissipation and is correlated strongly to the physiological state of the plant. The fluorescence signal recorded during illumination draws a characteristic induction curve with several inflection points, marked as *F*_o_, *F*_J_, *F*_I_ and *F*_M_. The induction curve is used for calculation of more than 30 parameters, which describe in great detail the efficiency of each step of the photosynthetic process.

The collected soil samples used in this study had a different nutrient constitution and all observed effects on the photosynthetic machinery were a consequence of the plants reaction to an environment formed by combinations of different nutrient deficiencies. Thus, the calculated fluorescent parameters showed changes in the photosynthetic process, measured in samples with varying contents, but with experimentally proven predominant deficiency (or presence) of a given macro- and microelement.

The results confirmed the negative effect of nutrient deficiency on the photosynthetic yield of PSII (Fig. 5). Nutrient deficiency induces some photo-inhibitory damage to PSII by a reduction in the quantum yield of PSII electron transport and the efficiency of the excitation of energy capture by open PSII reaction centres, *φ*_Po_, *φ*_Eo,_ *ψ*_Eo_. Similar results were obtained by Baker and Rosenqvist (2004) and Kalaji et al. (2014a). Moreover, in this study, the decrease in active reaction centres confirmed by the increase in ABS/RC was observed in all nutrient-deficient groups. This could be explained by the decrease in the number of active RCs (inactivation of RCs). The inactivation of reaction centres is considered to be a mechanism that protects the nutrient-deficient leaves against photo-oxidative damage and an excess of absorbed light energy (Kalaji et al. 2014a; Fig. 4, Supplementary Fig. S8).

Inspecting the differential curves between O and J confirmed the damage to the oxygen-evolving complex (OEC) visible as a strong increase in the K band as compared to the ‘control’ group. This is a sign of less-connected antennae complexes which can be due to improper membrane organisation and can lead to lower energy transfer and absorption efficiency. All groups with some specific nutrient deficiency showed positive O–K bands and the group experiencing the least amount of micro- and macroelements presented the highest band. Another effect is the band peak shift to the longer time periods, which can be due to slower energy transfer to the RCs.

A comparison of J–I normalised and I–P normalised curves demonstrated an imbalance between the reduction and oxidation of Q_A_ and the plastoquinone pool, respectively. The latter depends on the efficiency of electron uptake from the PSI acceptors and the number of available oxidised forms of NADP. Groups with moderate and strong deficiencies exhibit a negative band, which corresponds to a greater number of NADP+ molecules per active RC. This might be a compensatory mechanism developed by these plants in response to the suboptimal nutrient environment they were grown in.

Consecutive analysis of the element content in soil and plant leaf tissue at early growth stages and selected chlorophyll fluorescence parameters combined with PCA and novel machine-learning method—super-organising maps (sSOM)—is found to be a successful method for the early detection of plant stress resulting from a combination of nutrient deficiencies in natural conditions. Therefore, these results are very promising both in terms of research into the response of the photosynthetic apparatus to nutrient-deficiency stress and the regulatory processes in natural conditions, and for future application in agriculture.

## Conclusions

Plants develop different photosynthetic characteristics when subjected to varying environmental conditions such as variation in the availability of nutrients. These characteristics are traceable with the sensitive techniques developed for chlorophyll fluorescence measurement even when the overall physiological effect is still not visible.

The experimental set-up of this study mimicked real natural conditions in which plants were subjected to combinations of nutrient content and deficiencies. The macro- and microelement content of 60 different types of soil was determined and rapeseeds grown on these soils were studied—their leaf nutrient status was also defined. With these initial data, a full multi-step analysis was performed to see if a procedure could be established which allows the early determination of nutrient deficiency in rapeseeds only by using machine-learning methods based on data from the non-invasive in vivo measurement of chlorophyll *a* fluorescence.

The results confirmed that the combination of PCA, hierarchical k-means classification and super-organising maps can be a very informative tool to detect nutrient deficiency in early stages and even follow the changes that occur during senescence.

## Electronic supplementary material

Below is the link to the electronic supplementary material.

Supplementary material 1 (PDF 4410 KB)
