# Supplementary material for: Chlorophyll fluorescence as a tool for nutrient status identification in rapeseed plants
Source: Photosynth Res. 2017 Nov 28;136(3):329–43. doi: 10.1007/s11120-017-0467-7 (PMC5937862; doi:10.1007/s11120-017-0467-7)
Supplement: Supplementary file 1 — Supplementary material 1 (PDF 4410 KB) [file 11120_2017_467_MOESM1_ESM.pdf]

Supplementary information

Hazem M. Kalaji[1,2], Wojciech Bąba[3*], Krzysztof Gediga[4], Vasilij Goltsev [5], Izabela A. Samborska[1], Magdalena D. Cetner[1], Stella Dimitrova[5], Urszula Piszcz[4], Krzysztof Bielecki[4], Kamila Karmowska[4], Kolyo Dankov [5], Agnieszka Kompała-Bąba[6]

Chlorophyll fluorescence as a tool for nutrient status identification in rapeseed plants.
Photosynthesis Research xx: xx-xx.

Corresponding author: Wojciech Bąba e-mail: wojciech.baba12@gmail.com

Supplementary figures captions:

Supplemental Fig. S1. Graphical scheme representing the sequence of procedures performed in the described analysis.

Supplemental Fig. S2. Visualisation of cluster analyses of the soil data. The optimal number of cluster (4) was found with hkmeans method.

Supplemental Fig. S3. Visualisation of cluster analyses of the leaf element content after 25 DAS (upper dendrogram) and 40 DAS (lower dendrogram). The optimal number of clusters (3 and 4) was found with hkmeans method.

Supplemental Fig. S4. Results of the Principal Component Analysis of leaf micro- and macroelement contents in rapeseed leaves 25 days after sowing (25DAS). The four classes (marked with different colors). resulted from division of the third class (presented on Fig 2) into two ones.

Supplemental Fig. S5. Results of the Principal Component Analysis of leaf micro- and macroelement contents in rapeseed leaves 25 days after sowing (25DAS). Values of particular

elements (i.e. leaf Ca. Mg. N content. referred to size of the 'bubles' were plotted on the first two principal components (PCA. Dim.1 and Dim.2). Three groups were marked with different colors.

Supplemental Fig. S6. Results of the Principal Component Analysis of leaf micro- and macroelement contents in rapeseed leaves 25 days after sowing (25DAS). Values of particular elements (i.e. leaf Ca. Mg. N content. referred to size of the 'bubles' were plotted on the first two principal components (PCA. Dim.1 and Dim.2). Four groups were marked with different colors.

Supplemental Fig. S7. Results of the Principal Component Analysis of leaf micro- and macroelement contents in rapeseed leaves 40 days after sowing (40DAS). The four classes (marked with different colors). which resulted from hierarchical k-means classification algorithm were superimposed onto the graph.

Supplemental Fig. S8. Comparison of leaf micro- and macroelement contents in rapeseed leaves 40 days after sowing (40DAS). The values of particular element. referred to size of the 'bubles' were plotted on the first two principal components (PCA. Dim.1 and Dim.2). Four groups were marked with different colors.

Supplemental Fig. S9. Radar plot presenting the relative differences in Chlorophyll fluorescence parameters. in 5 groups distinguished in super SOM analysis.

Supplemental Fig. S10. Chlorophyll fluorescence transients in 5 groups distinguished in super SOM analysis

Supplemental Fig. S11. Correlations between the soil and leaf tissue 25 DAS (days after sowing) element contents. Three different methods of soil analysis was compared and soil with pH < 5.5. and >5.5 were analysed separately. The strength of correlations are marked with different colors (see. the color key). The grey colour means lack of data.

Supplemental_Figure S1

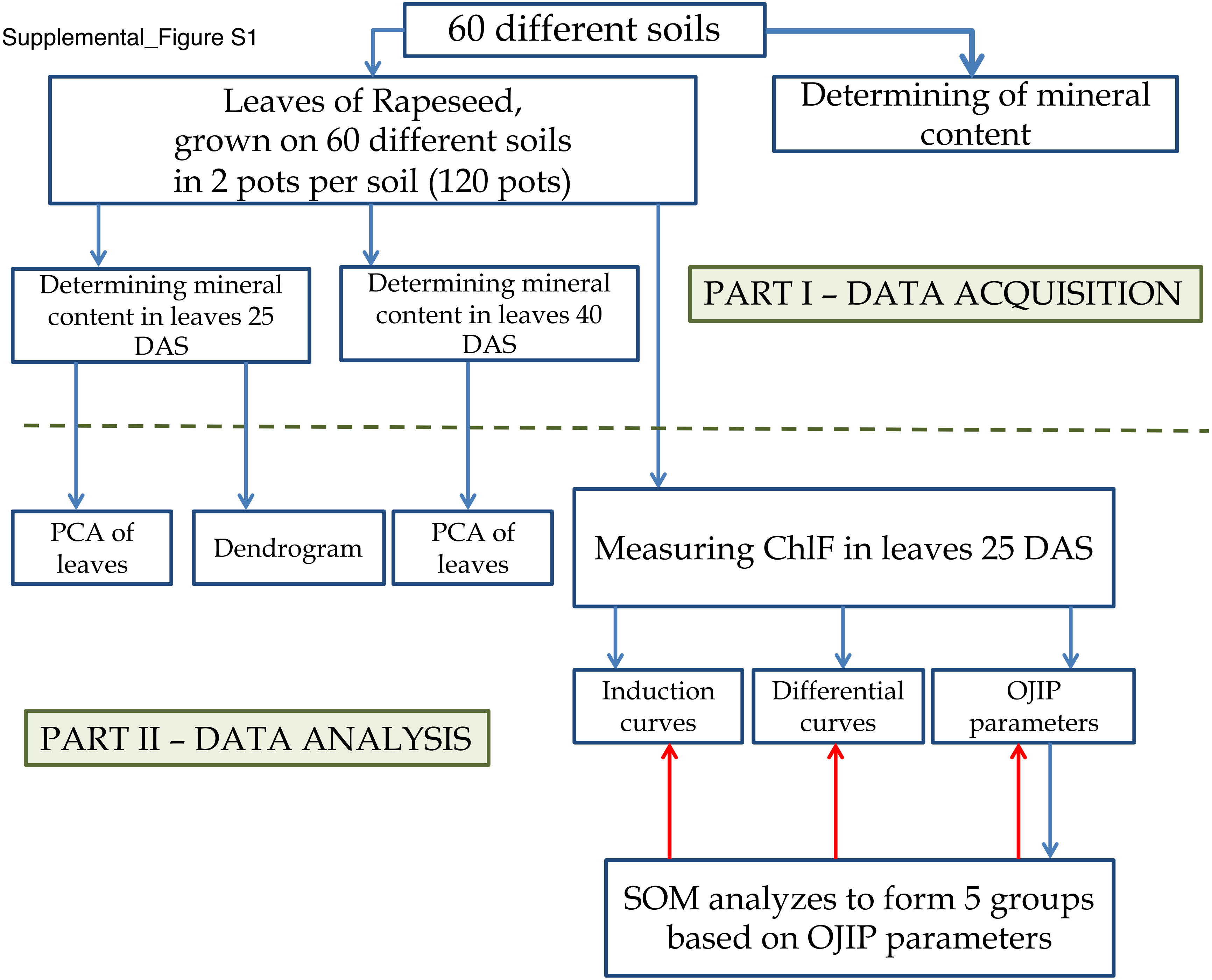

Supplemental_Figure S2.

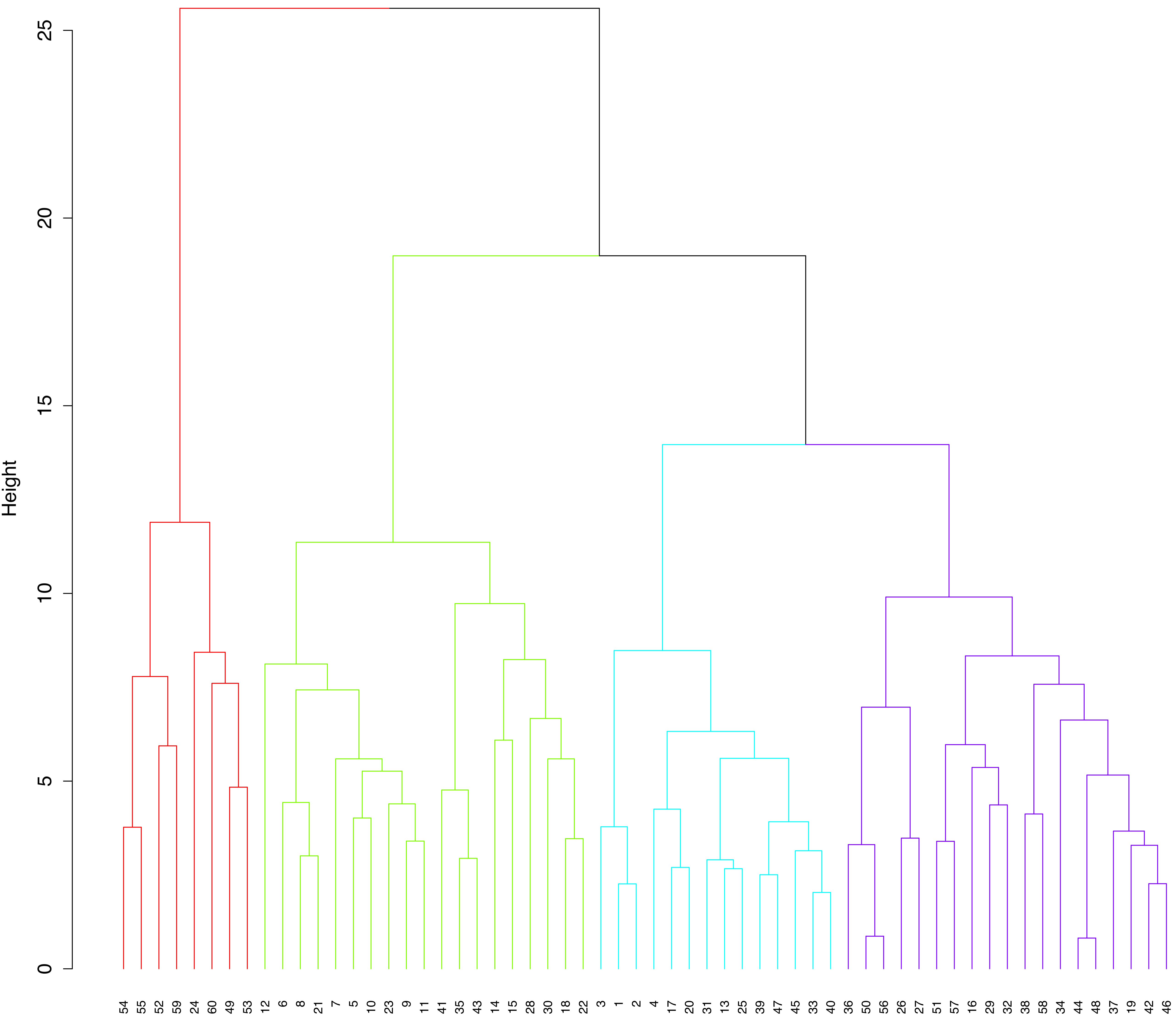

Supplemental_Figure S3.

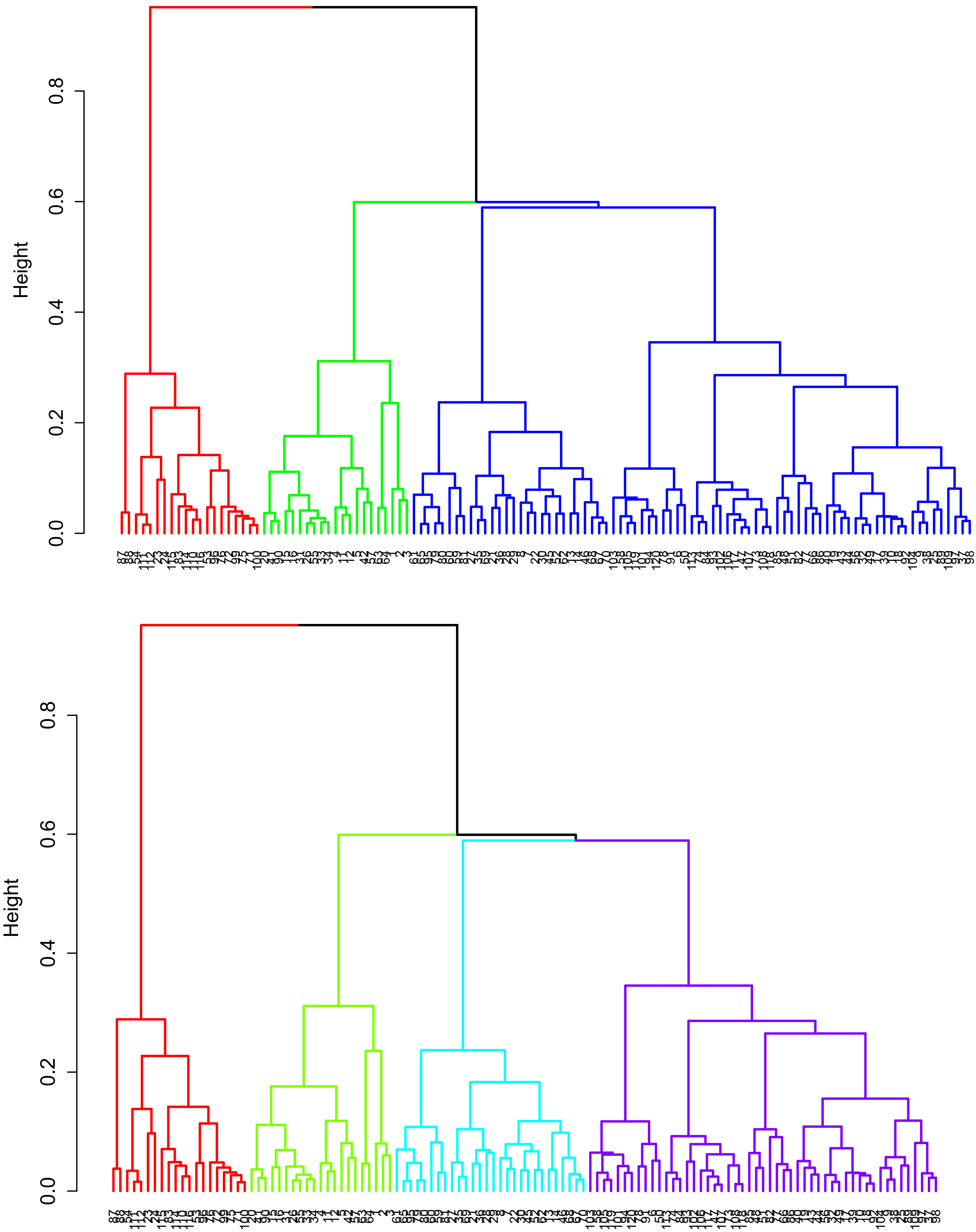

Supplemental_Figure S4.

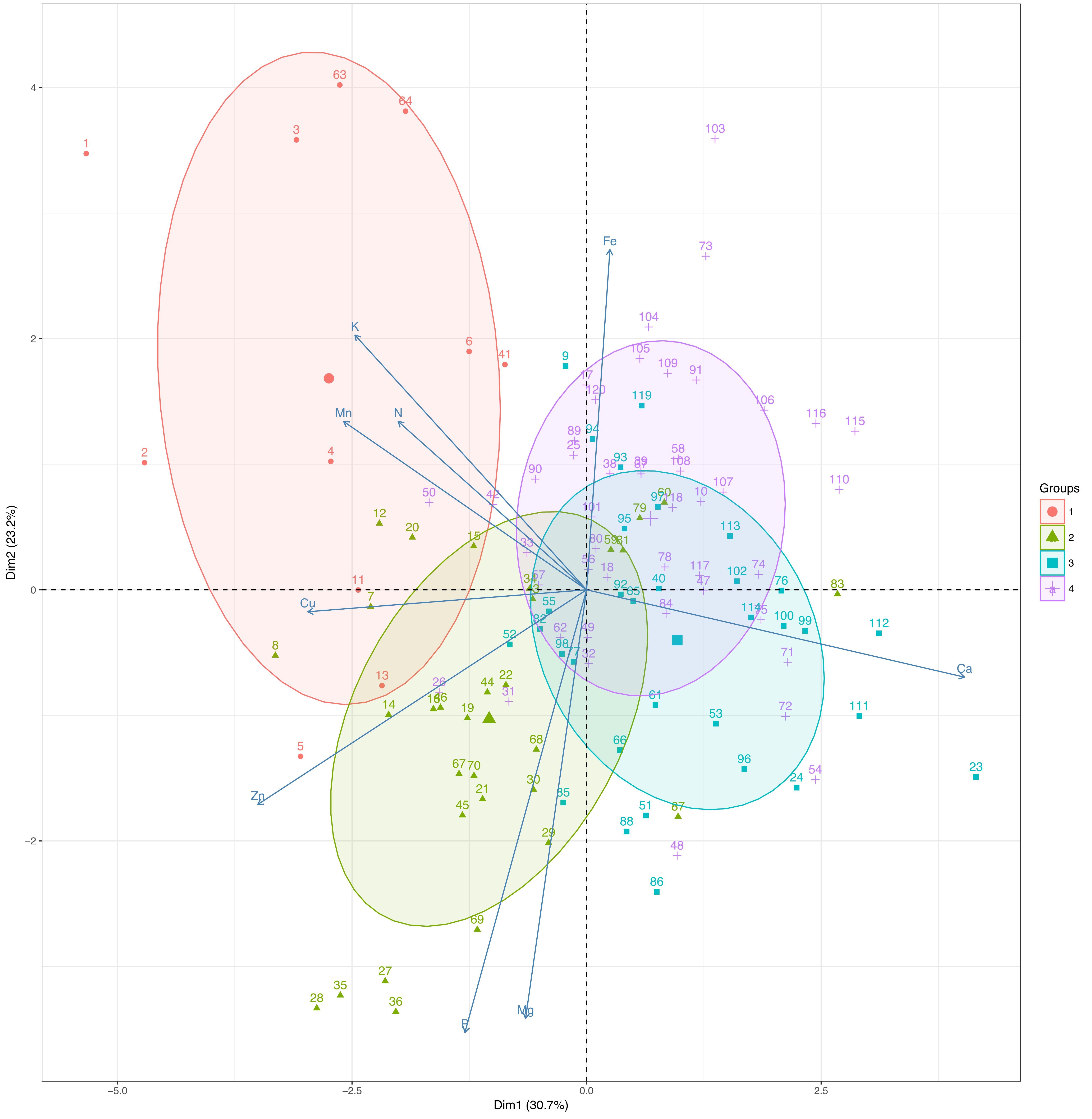

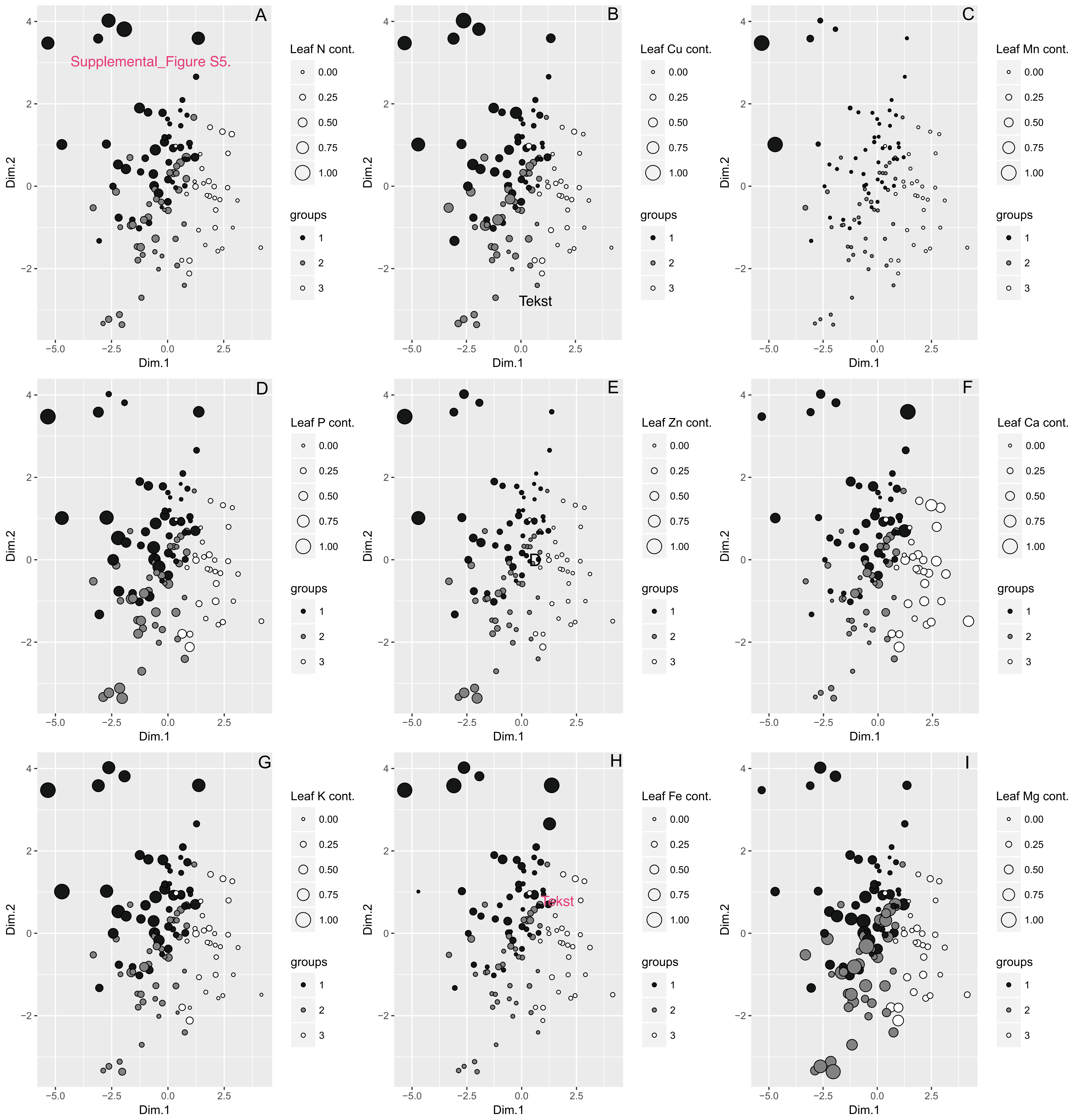
A
B
C
D
E
F
G
H
I
Supplemental_Figure S5.
Tekst
D
Tekst
Leaf N cont.
Leaf Cu cont.
Leaf Mn cont.
Leaf P cont.
Leaf Zn cont.
Leaf Ca cont.
Leaf K cont.
Leaf Fe cont.
Leaf Mg cont.
0.00
0.25
0.50
0.75
1.00
groups
1
2
3
Dim.1
Dim.2

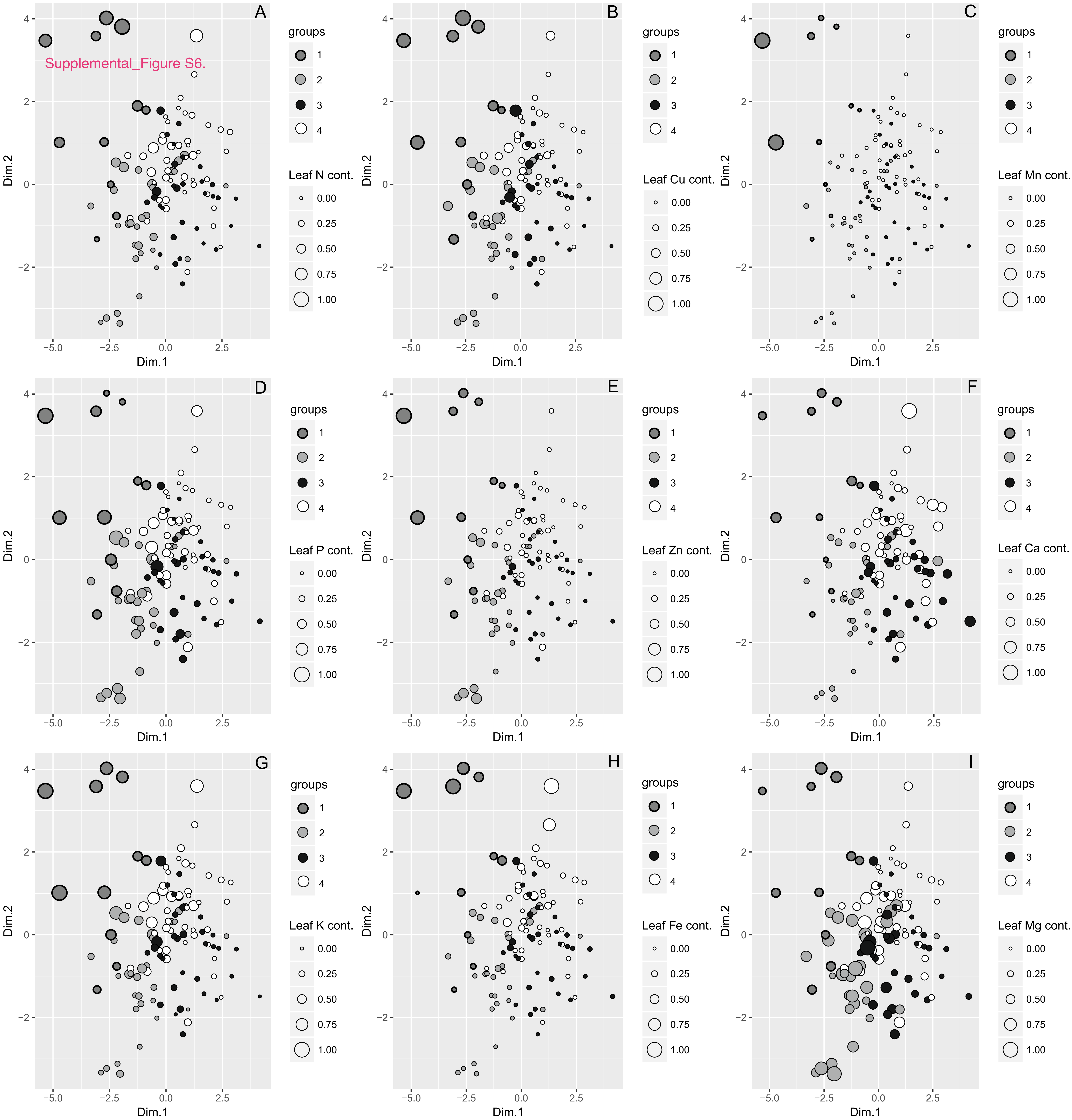
Supplemental_Figure S6.
A
B
C
D
E
F
G
H
I
Dim.1
Dim.2
groups
1
2
3
4
Leaf N cont.
Leaf Cu cont.
Leaf Mn cont.
Leaf P cont.
Leaf Zn cont.
Leaf Ca cont.
Leaf K cont.
Leaf Fe cont.
Leaf Mg cont.
0.00
0.25
0.50
0.75
1.00
−5.0
−2.5
0.0
2.5
−2
0
2
4

Supplemental_Figure S7.

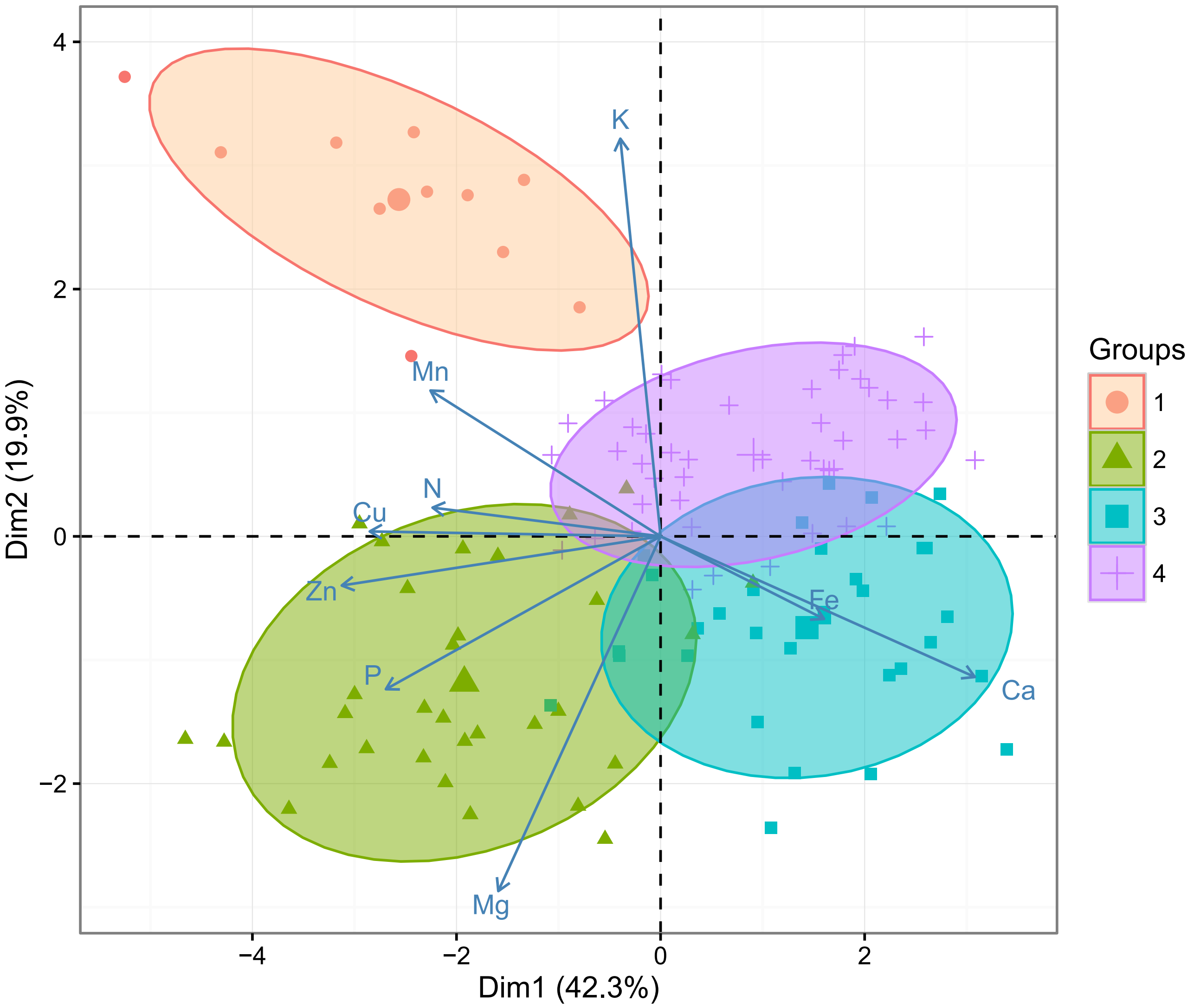

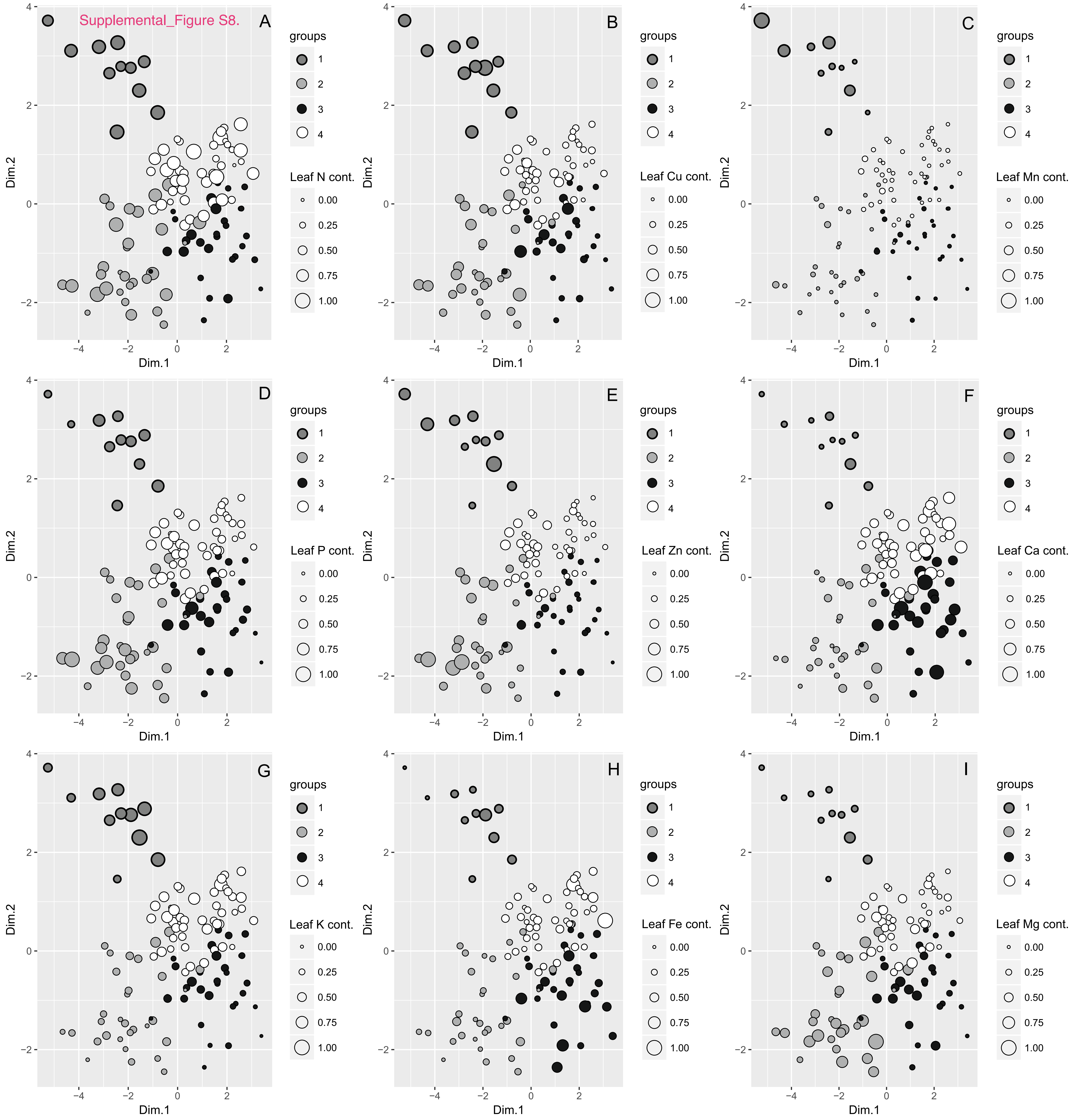

Supplemental_Figure S8.
A
B
C
D
E
F
G
H
I
groups
1
2
3
4
Leaf N cont.
Leaf Cu cont.
Leaf Mn cont.
Leaf P cont.
Leaf Zn cont.
Leaf Ca cont.
Leaf K cont.
Leaf Fe cont.
Leaf Mg cont.
0.00
0.25
0.50
0.75
1.00
Dim.1
Dim.2

Supplemental_Figure S9.

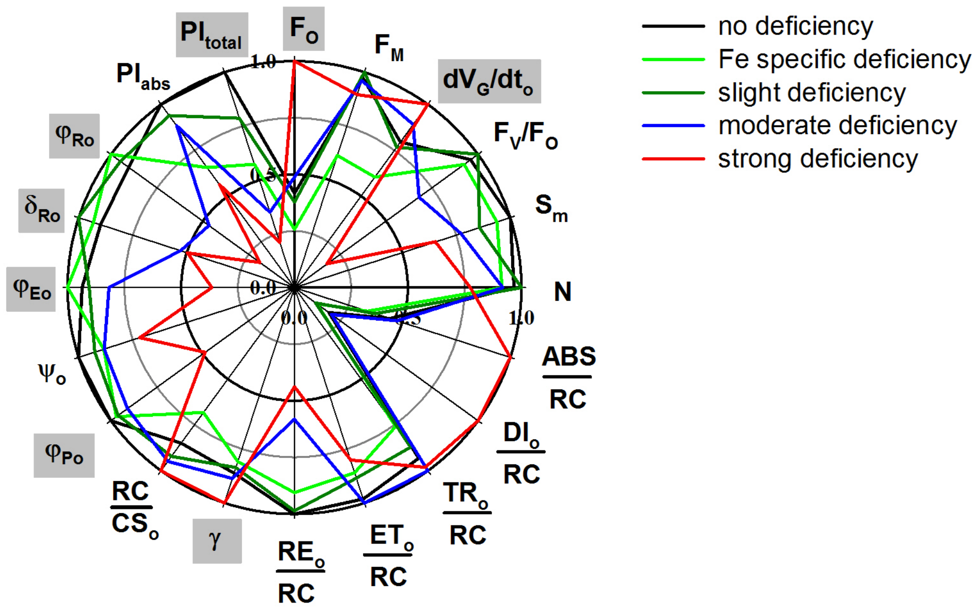

Supplemental_Figure S10.

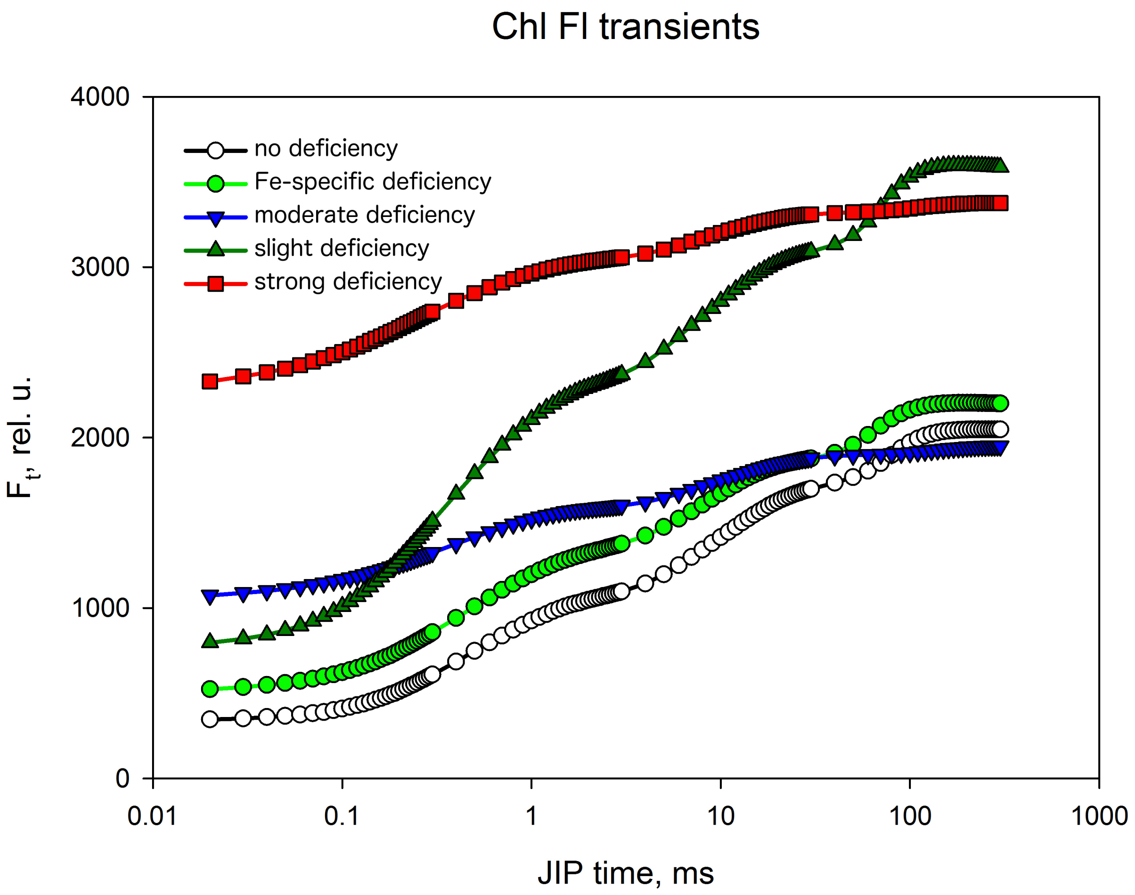

Supplemental_Figure S11.

A. Correlations between soil and plant 25 DAS element content and (OSCh method) for soil pH < 5.5. The strenght of correlations are marked with colors (see. the color key). Grey - no data

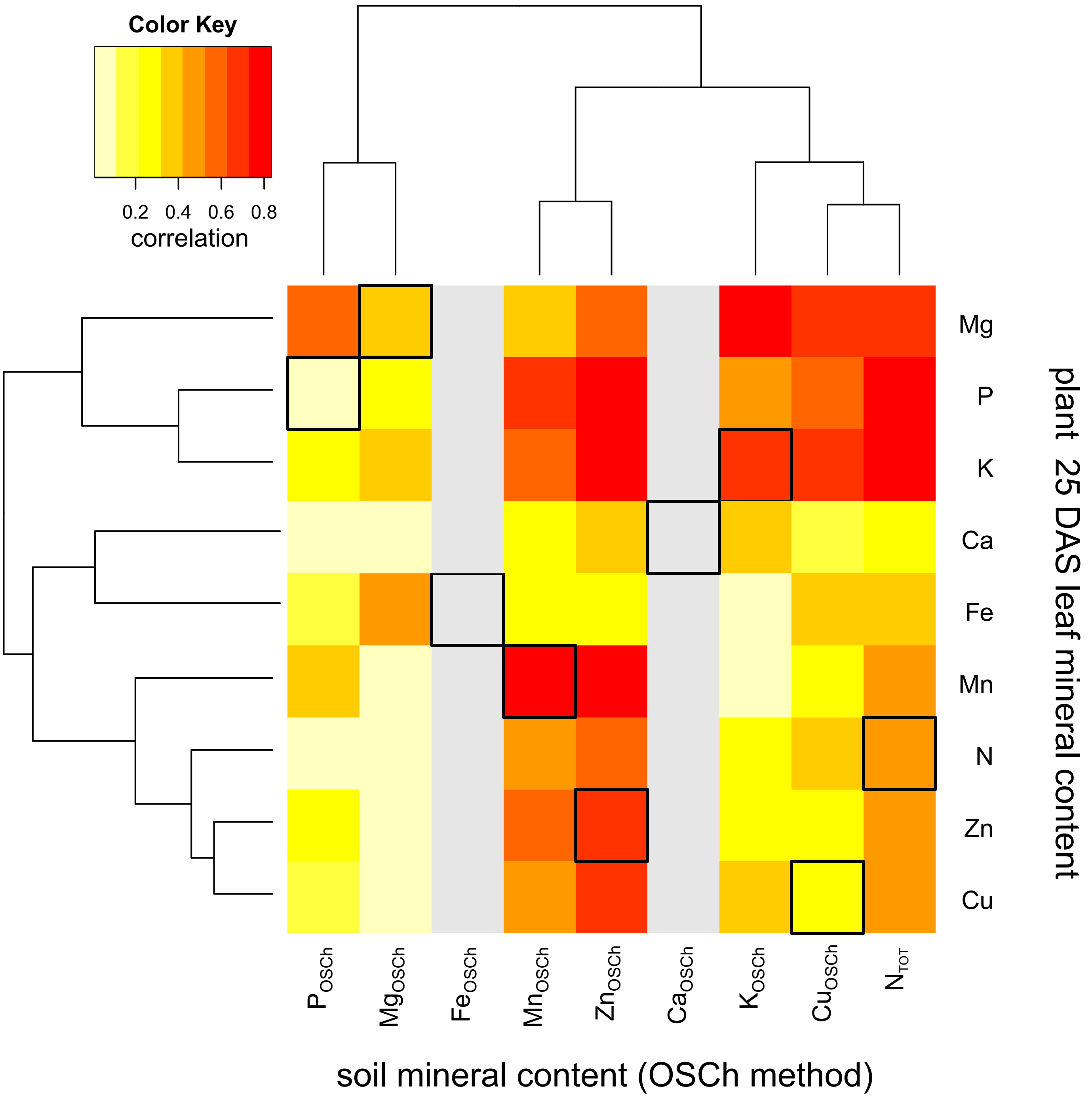

Supplemental_Figure S11

B. Correlations between soil and plant 25 DAS element content and (Mehlich method) for soil pH < 5.5. The strenght of correlations are marked with colors (see. the color key). Grey - no data

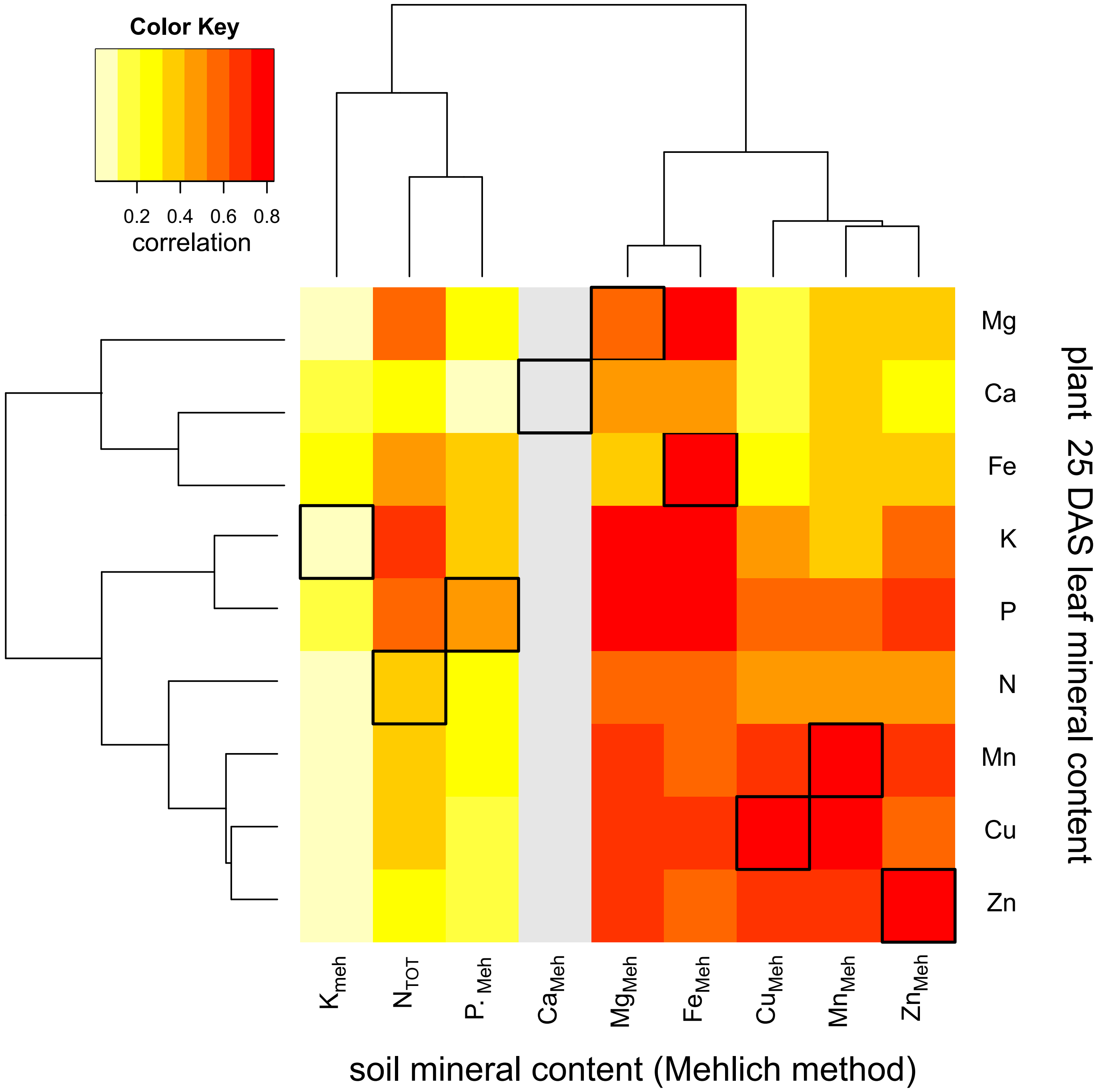

Supplemental_Figure S11

C. Correlations between soil and plant 25 DAS element content and (Mehlich method) for soil pH < 5.5. The strenght of correlations are marked with colors (see. the color key). Grey - no data

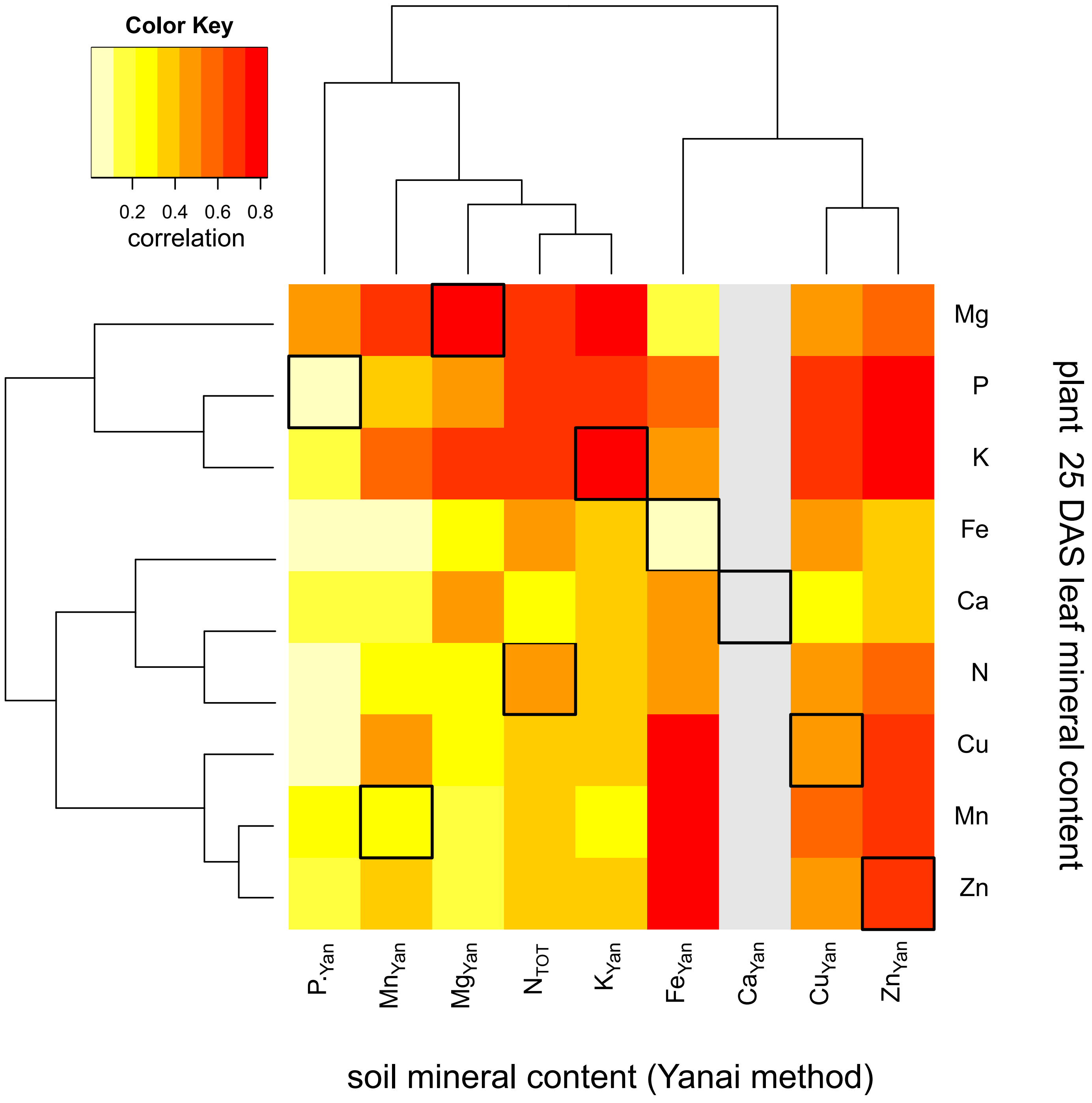

Supplemental_Figure S11

D. Correlations between soil and plant 25 DAS element content and (OSCh method) for soil pH > 5.5. The strenght of correlations are marked with colors (see. the color key). Grey - no data

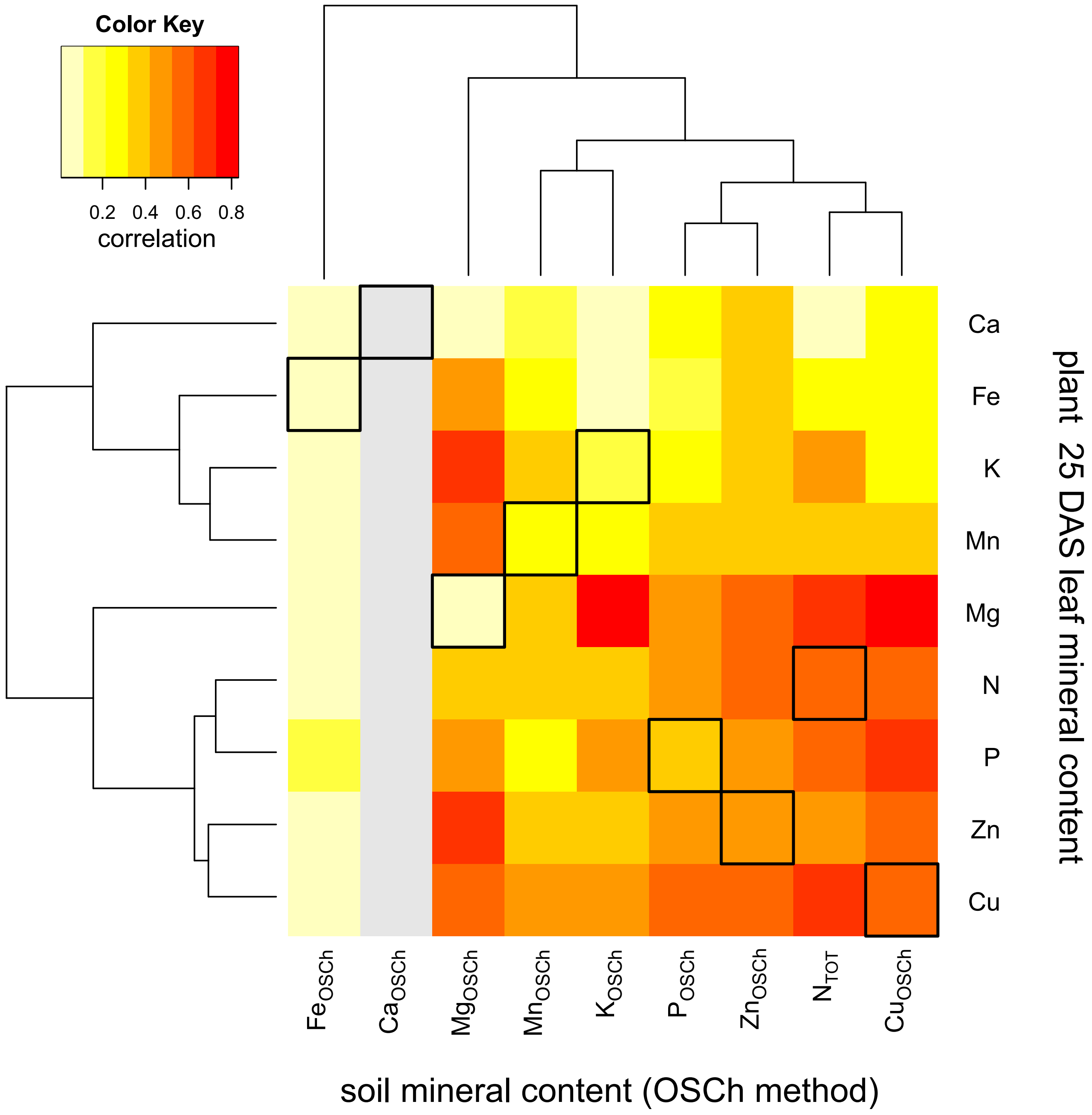

Supplemental_Figure S11

E. Correlations between soil and plant 25 DAS element content and (Mehlich method) for soil pH > 5.5. The strenght of correlations are marked with colors (see. the color key). Grey - no data

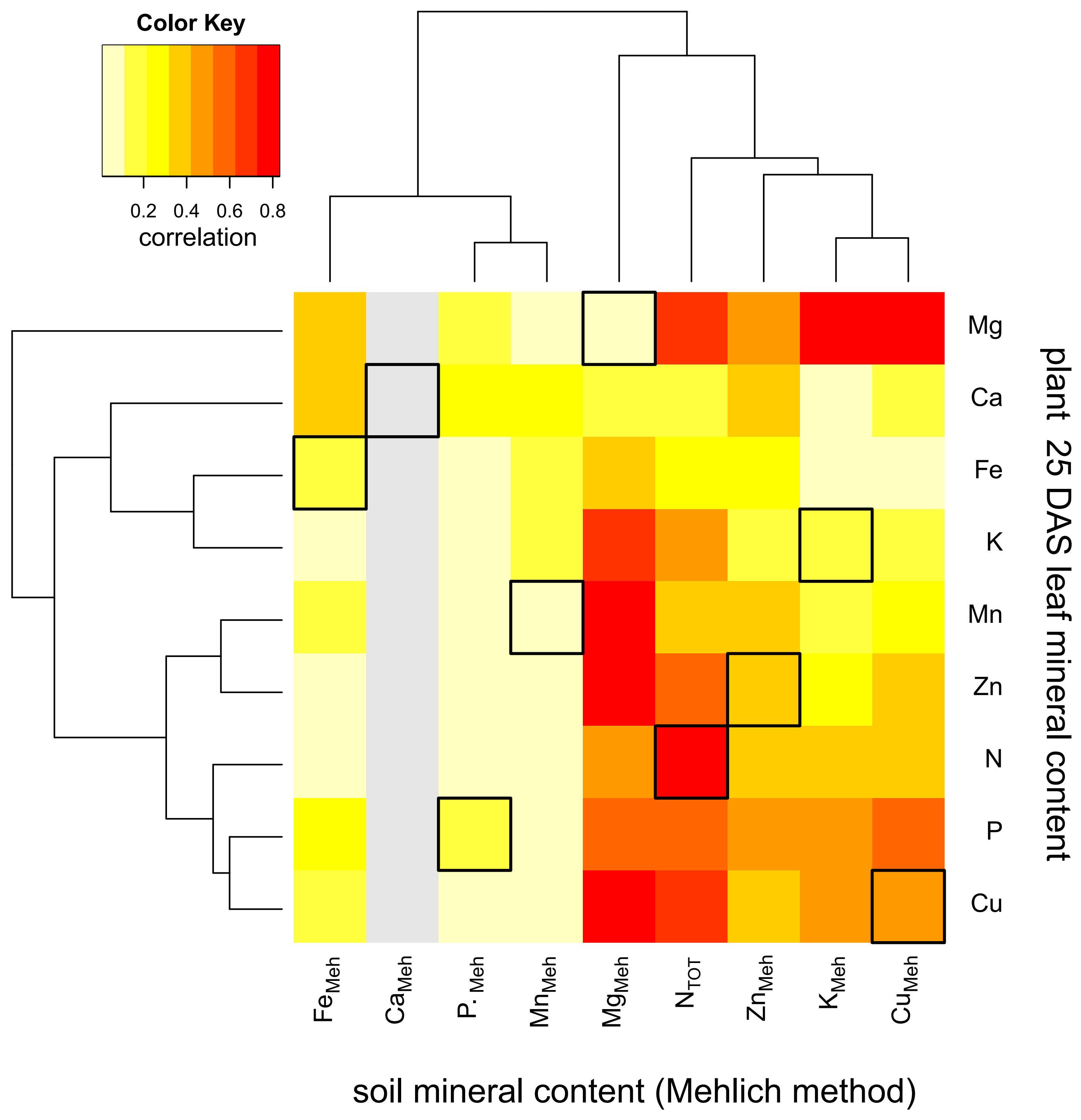

Supplemental_Figure S11

F. Correlations between soil and plant 25 DAS element content and (Yanai method) for soil pH > 5. The strenght of correlations are marked with colors (see. the color key). Grey - no data

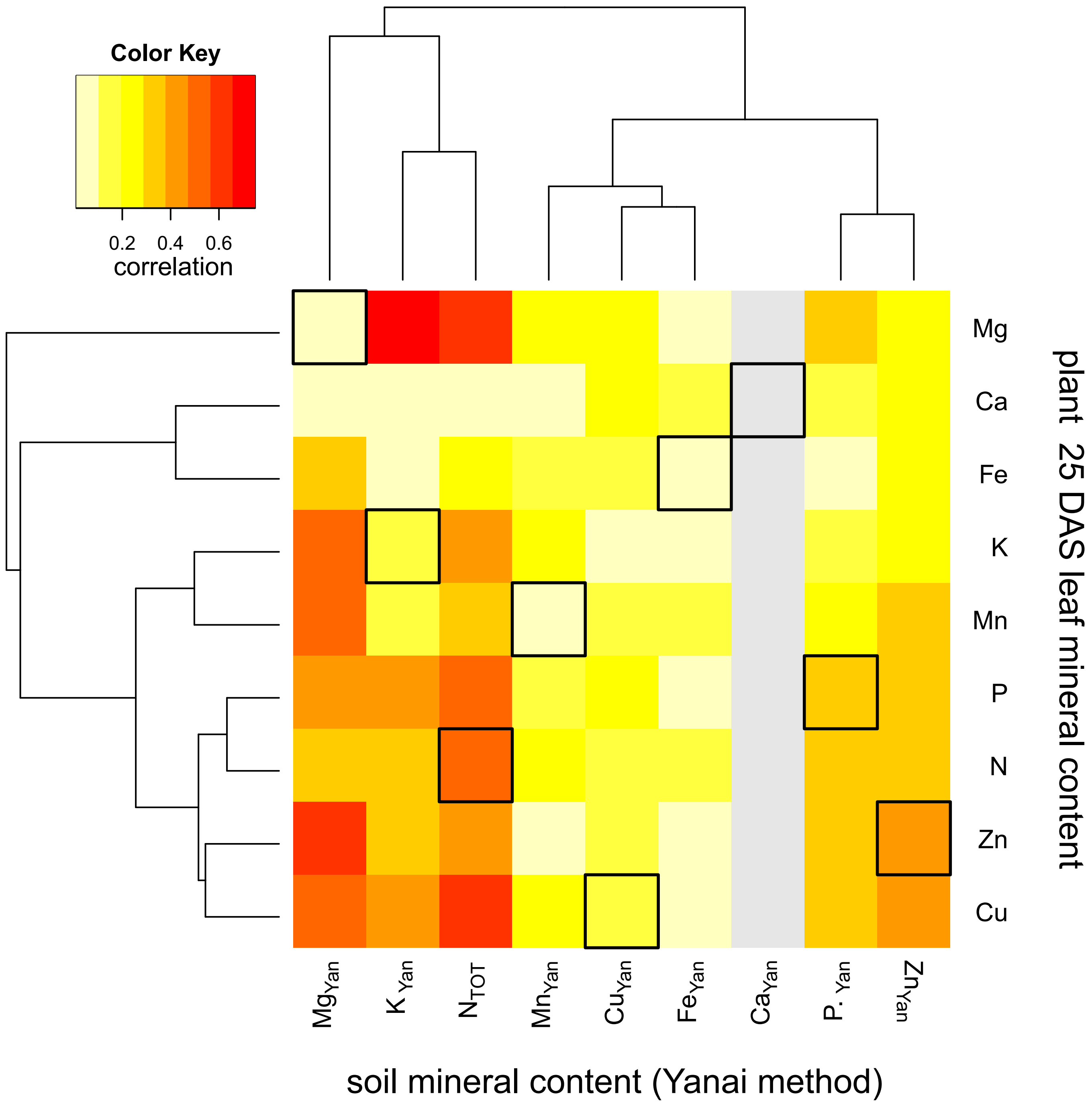

Supplemental Table S1. Classification of soil samples used in the experiment. The average values with standard errors of the selected physico-chemical soil properties were presented. The values with the same letters were not significantly different at $p < 0.05$, according to post-hoc Tukey honest difference test. The three different method of soil analysis were compared: osh – Polish norm, meh – Mehrih 3 method, yan – Yanai method.

class	pH		fine particles		clay		osh_Cu		osh_Zn		osh_Mn		osh_C.org
1	6,76 ± 0,64	a	31,5 ± 7,92	a	11,25 ± 2,2	a	17,25 ± 1,23	a	46,8 ± 5,07	a	212,71 ± 42,14	a	2167,75 ± 221,75
2	6,32 ± 0,39	a	27,7 ± 3,5	a	7,78 ± 1,92	b	8,38 ± 1,14	b	29,74 ± 9,31	b	184,63 ± 64,01	a	1415,07 ± 610,71
3	5,73 ± 0,45	b	16,87 ± 9,51	b	5,47 ± 2,52	bc	4,74 ± 5,94	bc	17,3 ± 4,33	c	120,39 ± 76,33	b	1177,04 ± 325,68
4	5,37 ± 0,4	b	10,43 ± 5,42	b	3,86 ± 4,53	c	3,62 ± 3,44	c	13,13 ± 14,95	c	106,93 ± 61,94	b	769,2 ± 692,29

class	osh_Ntot		osh_Hh		osh_K		osh_P		osh_Mg		meh_K		meh_P
1	182,62 ± 22,36	a	26,55 ± 10,05	a	498,5 ± 59,77	a	306,5 ± 37,59	a	101,93 ± 28,07	a	206,88 ± 24,22	a	328,93 ± 112,6
2	127,86 ± 51,84	b	22,81 ± 8,81	a	245,39 ± 71,33	b	278,71 ± 107,69	a	99,91 ± 26,26	a	108,13 ± 49,61	b	230,25 ± 141,37
3	124,48 ± 30,78	b	13,1 ± 5,07	b	190,07 ± 71,62	bc	149,78 ± 46,38	b	81,38 ± 33,07	ab	88,5 ± 62,58	b	220,2 ± 47,02
4	75,93 ± 55,3	c	8,99 ± 5,39	b	150,47 ± 95,37	c	105,87 ± 104,36	b	54,8 ± 26,74	b	63,2 ± 97,04	b	181,48 ± 83,18

class	meh_Mg		meh_Mn		meh_Fe		meh_Zn		meh_Cu		yan_K		yan_P
1	199,12 ± 32,99	a	101,38 ± 29,17	a	373,14 ± 68,61	a	26,54 ± 4,43	a	10,51 ± 1,23	a	333,62 ± 26,66	a	281,25 ± 52,21
2	181,07 ± 48,71	a	96,39 ± 24,37	a	342 ± 67,95	ab	21,44 ± 9,21	a	4,3 ± 3,13	b	112,3 ± 38,69	b	277,71 ± 109,34
3	177,87 ± 32,5	a	92,73 ± 29,77	a	336,83 ± 78,88	ab	11,8 ± 4,94	b	4,29 ± 2,43	b	61,21 ± 69,01	b	136,61 ± 37,21
4	142,13 ± 39,23	b	80,14 ± 35,09	a	269,88 ± 59,54	b	10,03 ± 10,8	b	3,15 ± 3,74	b	56,93 ± 151,02	b	131,33 ± 111,46

class	yan_Mg		yan_Mn		yan_Fe		yan_Zn		yan_Cu	
1	267,07 ± 56,21	a	523,25 ± 108,44	a	153,29 ± 43,9	a	26,84 ± 4,03	a	4,59 ± 0,55	a
2	243 ± 116,96	ab	450,04 ± 150,95	a	90,93 ± 61,8	b	22,64 ± 9,31	a	2,88 ± 1,08	b
3	232,57 ± 57,64	ab	329,33 ± 131	b	67,83 ± 29,65	b	9,88 ± 3,74	b	2,72 ± 0,86	b
4	150,13 ± 65,86	b	316,43 ± 154,19	b	66,00 ± 25,78	b	8,03 ± 15,42	b	2,5 ± 3,22	b

Supplemental Table S2. Classification of selected micro- and macroelement contents in rapeseed leaves after 25 and 40 days after sowing (25 and 40DAS respectively). The groups were distinguished according to Ward method of hierarchical clustering with Euclidean distance (the details were on Fig. 1). The means ± SE for groups were presented. The values with the same letters were not significantly different at $p < 0.05$, according to Tukey honest difference test.

	class	N [g/kg]		P [g/kg]		K [g/kg]		Ca [g/kg]		Mg [g/kg]	
25 DAS	1	22,87 ± 10,13	a	4,94 ± 1,73	a	25,24 ± 9,89	a	16,87 ± 4,30	a	7,05 ± 1,95	a
	2	19,26 ± 4,36	b	4,54 ± 1,00	a	15,97 ± 4,14	b	15,10 ± 2,69	ab	5,56 ± 1,81	b
	3	12,87 ± 2,74	c	3,37 ± 0,76	b	11,79 ± 3,54	c	13,33 ± 3,31	b	4,10 ± 1,19	c
	1	32,45 ± 11,33	a	6,15 ± 1,76	a	34,50 ± 9,18	a	15,84 ± 3,36	a	7,57 ± 0,87	a
	2	21,09 ± 5,41	b	5,04 ± 1,20	a	18,76 ± 7,25	b	15,81 ± 2,86	ab	6,19 ± 1,63	ab
	3	18,24 ± 4,71	bc	4,14 ± 1,04	b	18,38 ± 6,13	b	14,82 ± 3,19	ab	4,91 ± 1,87	b
	4	14,06 ± 6,80	c	3,61 ± 1,27	b	13,82 ± 7,59	b	13,48 ± 4,56	b	4,86 ± 1,80	b
40 DAS	1	23,78 ± 6,81	a	7,87 ± 1,89	a	29,39 ± 9,06	a	29,79 ± 7,50	a	12,30 ± 2,79	a
	2	22,75 ± 5,85	a	7,15 ± 2,05	a	21,68 ± 5,98	b	28,98 ± 8,61	a	9,17 ± 3,25	b
	3	18,38 ± 5,73	b	5,55 ± 1,51	b	20,37 ± 6,94	b	27,91 ± 6,86	a	7,77 ± 1,71	b
	1	28,67 ± 3,09	a	8,29 ± 1,49	a	38,58 ± 9,44	a	31,96 ± 6,00	a	12,62 ± 2,68	a
	2	23,93 ± 6,07	a	8,10 ± 2,17	a	27,79 ± 6,03	b	31,66 ± 5,67	a	9,08 ± 3,41	b
	3	23,23 ± 3,81	a	6,78 ± 1,89	ab	20,62 ± 5,86	c	23,42 ± 7,93	b	8,72 ± 2,66	b
	4	16,41 ± 6,35	b	5,79 ± 1,65	b	18,95 ± 6,08	c	22,75 ± 6,30	b	8,72 ± 2,63	b

	class	Cu [mg/kg]				Fe [mg/kg]				Mn [mg/kg]				Zn [mg/kg]			
25 DAS	1	20,00	±	2,00	a	30,00	±	6,00	a	130,00	±	17,00	a	40,00	±	26,00	a
	2	20,00	±	6,00	a	20,00	±	7,00	b	30,00	±	8,00	a	40,00	±	17,00	a
	3	10,00	±	2,00	b	20,00	±	4,00	b	20,00	±	8,00	a	20,00	±	6,00	b
	1	36,43	±	9,48	a	49,18	±	28,61	a	492,61	±	69,18	a	72,80	±	32,17	a
	2	22,35	±	6,70	b	27,57	±	8,36	b	35,08	±	11,59	b	43,72	±	18,17	b
	3	17,11	±	6,78	c	25,11	±	6,68	b	21,66	±	12,10	b	26,20	±	7,23	c
	4	16,41	±	5,39	c	20,00	±	15,95	b	19,84	±	16,92	b	21,08	±	10,59	c
40 DAS	1	5,68	±	1,93	a	35,30	±	10,76	a	66,53	±	18,74	a	76,49	±	31,90	a
	2	5,53	±	1,48	a	32,82	±	9,68	a	44,84	±	25,83	ab	68,44	±	34,21	a
	3	3,78	±	0,95	b	30,29	±	14,31	a	32,04	±	13,73	b	42,56	±	15,51	b
	1	8,39	±	0,85	a	35,19	±	15,47	a	172,48	±	12,45	a	99,37	±	34,67	a
	2	5,71	±	1,49	b	35,14	±	7,47	ab	44,11	±	25,46	b	86,51	±	36,37	a
	3	4,60	±	1,49	c	33,97	±	12,20	ab	36,67	±	17,78	b	56,11	±	14,32	b
	4	4,25	±	1,16	c	26,97	±	11,22	b	35,72	±	19,89	b	43,83	±	21,14	b

Supplemental Table S3. Comparison of values of selected measured and calculated chlorophyll *a* fluorescence parameters (ChlF) in rapeseed plants at 25 DAS groups differed in leaf micro- and macroelement. The means ± SE for three groups were presented. The values with the same letters were not significantly different at $p < 0.05$, according to Tukey honest difference test. The list of chlorophyll fluorescence parameters is presented in Supplemental Table S6.

	Group											
	1				2				3			
Fo	709,32	±	32,51	a	672,64	±	34,42	b	867,22	±	51,01	b
Fm	2812,09	±	128,89	a	2786,74	±	142,58	a	2709,58	±	159,39	a
Fv	2102,77	±	96,38	a	2114,10	±	108,17	a	1842,36	±	108,37	b
Fv/Fo	3,49	±	0,16	a	3,63	±	0,19	a	2,62	±	0,15	b
dV/dto	0,99	±	0,05	a	0,96	±	0,05	a	1,10	±	0,06	b
Sm	15,74	±	0,72	a	16,44	±	0,84	b	14,70	±	0,86	c
N	29,71	±	1,36	a	30,79	±	1,58	b	28,82	±	1,70	c
Vj	0,51	±	0,02	a	0,50	±	0,03	b	0,54	±	0,03	c
Vi	0,86	±	0,04	a	0,85	±	0,04	a	0,89	±	0,05	b
ABS/RC	2,78	±	0,13	a	2,72	±	0,14	a	3,51	±	0,21	b
TRo/RC	1,92	±	0,09	a	1,91	±	0,10	b	2,02	±	0,12	b
ETo/RC	0,93	±	0,04	a	0,95	±	0,05	b	0,92	±	0,05	a
DIo/RC	0,86	±	0,04	a	0,81	±	0,04	a	1,49	±	0,09	b
REo/RC	0,27	±	0,01	a	0,28	±	0,01	a	0,22	±	0,01	b
phi/Po	0,74	±	0,03	a	0,75	±	0,04	a	0,67	±	0,04	b
psi/Eo	0,49	±	0,02	a	0,50	±	0,03	b	0,46	±	0,03	c
phi/Eo	0,37	±	0,02	a	0,39	±	0,02	b	0,32	±	0,02	c
deltaRo	0,29	±	0,01	a	0,29	±	0,02	a	0,24	±	0,01	b
phiRo	0,11	±	0,01	a	0,12	±	0,01	a	0,08	±	0,00	b
PI.abs	8,98	±	0,41	a	8,58	±	0,44	a	8,41	±	0,49	a
PI.total	4,18	±	0,19	a	3,60	±	0,18	ab	2,69	±	0,16	b
RC/Cso	253,78	±	11,60	ab	241,74	±	12,58	b	245,62	±	15,39	a
GAMMA_RC	0,39	±	0,03	a	0,40	±	0,04	a	0,34	±	0,04	b
tFM	217,0798	±	9,949838	a	221,623	±	11,339221	b	252,0069	±	9,823936	b

Supplemental Table S4. Comparison of values of selected measured and calculated chlorophyll *a* fluorescence parameters (ChlF) in rapeseed plants at 25 DAS groups differed in leaf micro- and macroelement The means ± SE for four groups were presented. The values with the same letters were not significantly different at $p < 0.05$, according to Tukey honest difference test.

	Group											
	1				2				3				4			
Fo	684,57	±	68,80	b	691,11	±	39,32	b	778,78	±	44,96	a	752,28	±	35,90	ab
Fm	2989,09	±	300,41	a	2828,51	±	160,91	ab	2784,76	±	160,78	bc	2689,74	±	128,37	c
Fv	2304,53	±	231,61	a	2137,40	±	121,59	ab	2005,98	±	115,82	bc	1937,46	±	92,47	c
Fv.Fo	3,77	±	0,38	a	3,57	±	0,20	a	3,10	±	0,18	b	3,18	±	0,15	b
dV.dto	0,95	±	0,10	a	0,96	±	0,05	b	1,08	±	0,06	b	1,01	±	0,05	b
Sm	16,47	±	1,66	a	16,43	±	0,93	a	15,12	±	0,87	b	15,45	±	0,74	b
N	30,15	±	3,03	ab	30,77	±	1,75	a	29,74	±	1,72	b	29,20	±	1,39	b
Vj	0,51	±	0,05	ab	0,50	±	0,03	a	0,52	±	0,03	b	0,52	±	0,02	b
Vi	0,83	±	0,08	a	0,84	±	0,05	b	0,88	±	0,05	c	0,87	±	0,04	d
ABS.RC	2,47	±	0,25	a	2,72	±	0,15	a	3,06	±	0,18	ab	3,13	±	0,15	b
TRo.RC	1,86	±	0,19	a	1,91	±	0,11	a	2,03	±	0,12	b	1,93	±	0,09	a
ETo.RC	0,91	±	0,09	a	0,95	±	0,05	b	0,95	±	0,05	b	0,92	±	0,04	a
DIo.RC	0,61	±	0,06	a	0,82	±	0,05	a	1,03	±	0,06	b	1,20	±	0,06	b
REo.RC	0,31	±	0,03	a	0,29	±	0,02	a	0,23	±	0,01	b	0,25	±	0,01	c
phi.Po.	0,77	±	0,08	a	0,75	±	0,04	a	0,72	±	0,04	b	0,71	±	0,03	b
psi.Eo.	0,49	±	0,05	ab	0,50	±	0,03	a	0,48	±	0,03	b	0,48	±	0,02	b
phi.Eo.	0,38	±	0,04	a	0,38	±	0,02	a	0,35	±	0,02	b	0,35	±	0,02	b
delta.Ro.	0,34	±	0,03	a	0,30	±	0,02	b	0,25	±	0,01	c	0,27	±	0,01	d
phi.Ro.	0,13	±	0,01	a	0,12	±	0,01	a	0,09	±	0,01	b	0,10	±	0,00	c
PI.abs	9,06	±	0,91	a	8,47	±	0,48	a	9,08	±	0,52	a	8,53	±	0,41	a
PI.total	4,83	±	0,49	a	3,76	±	0,21	ab	3,60	±	0,21	ab	3,25	±	0,16	b
RC.ABS	0,42	±	27,11	a	0,40	±	14,37	a	0,36	±	14,61	b	0,38	±	11,89	b

GAMMA_RC	0,71	±	0,07	a	0,72	±	0,04	a	0,74	±	0,04	b	0,73	±	0,03	b
tFM	204,3434343	±	20,54	a	217,9288026	±	12.39754	b	226,45	±	14,21	bc	246,13	±	10,81	c

Supplemental Table S5. Comparison of values of selected measured and calculated chlorophyll *a* fluorescence parameters (ChlF) in rapeseed plants at 40 DAS groups differed in leaf micro- and macroelement. The means ± SE for four groups were presented. The values with the same letters were not significantly different at $p < 0.05$, according to Tukey honest difference test.

	Group															
	1				2				3				4			
Fo	865,33	±	87,41	a	801,85	±	46,61	a	825,03	±	47,63	a	807,41	±	39,30	a
Fm	2985,35	±	301,57	a	2901,02	±	168,62	a	2707,29	±	156,31	b	2668,71	±	129,91	b
Fv	2120,02	±	214,15	a	2099,17	±	122,01	a	1882,26	±	108,67	b	1861,30	±	90,61	b
FvFo	3,27	±	0,33	a	3,21	±	0,19	a	2,80	±	0,16	b	2,88	±	0,14	b
dV.dto	1,13	±	0,11	a	1,17	±	0,07	a	1,34	±	0,08	b	1,26	±	0,06	c
Sm	14,47	±	1,46	a	14,47	±	0,84	a	14,79	±	0,85	a	14,50	±	0,71	a
N	26,85	±	2,71	c	28,61	±	1,66	bc	31,91	±	1,84	a	29,17	±	1,42	b
Vj	0,58	±	0,06	ab	0,57	±	0,03	a	0,58	±	0,03	ab	0,59	±	0,03	b
Vi	0,86	±	0,09	b	0,87	±	0,05	bc	0,88	±	0,05	a	0,88	±	0,04	a
ABS.RC	3,09	±	0,31	bc	3,07	±	0,18	c	3,70	±	0,21	a	3,49	±	0,17	ab
TRo.RC	1,92	±	0,19	c	2,02	±	0,12	bc	2,26	±	0,13	a	2,08	±	0,10	b
ETo.RC	0,79	±	0,08	c	0,85	±	0,05	b	0,92	±	0,05	a	0,82	±	0,04	bc
DIo.RC	1,17	±	0,12	ab	1,05	±	0,06	b	1,44	±	0,08	a	1,40	±	0,07	a
REo.RC	0,26	±	0,03	a	0,25	±	0,01	a	0,24	±	0,01	a	0,24	±	0,01	a

phiPo	0,70	± 0,07	ab	0,72	± 0,04	a	0,68	± 0,04	ab	0,68	± 0,03	b
psiEo	0,42	± 0,04	ab	0,43	± 0,02	a	0,42	± 0,02	ab	0,41	± 0,02	b
phiEo	0,31	± 0,03	ab	0,31	± 0,02	a	0,29	± 0,02	ab	0,29	± 0,01	b
deltaRo	0,31	± 0,03	a	0,29	± 0,02	b	0,27	± 0,02	c	0,28	± 0,01	b
phiRo	0,11	± 0,01	a	0,10	± 0,01	ab	0,08	± 0,00	c	0,09	± 0,00	bc
PI.abs	10,79	± 1,09	a	11,54	± 0,67	a	11,62	± 0,67	a	12,20	± 0,59	a
PI.total	5,01	± 0,51	a	4,73	± 0,27	a	4,38	± 0,25	a	4,82	± 0,23	a
RC.ABS	0,38	± 28,29	a	0,36	± 15,13	ab	0,32	± 13,47	c	0,34	± 11,78	b
GAMMA_RC	0,73	± 0,07	a	0,74	± 0,04	a	0,76	± 0,04	b	0,75	± 0,04	c
tFM	222,55	± 22,48	c	233,37	± 13,56	bc	248,46	± 14.34	a	240,54	± 11,71	ab

Supplemental Table S6. Summary of measured and calculated Chl *a* fluorescence. Based on Srivastava et al. 1995, Strasser et al. 2004, Tsimilli-Michael and Strasser 2008, Strasser et al. 2010).

**Fluorescence parameter**	**Description**
**Measured parameters and basic JIP-test parameters derived from the OJIP transient**	
$F_O = F_{20\,\mu s}$	Minimum fluorescence, when all PSII reaction centers (RCs) are open, Fluorescence intensity at 20 µs
$F_J = F_{2ms}$	Fluorescence intensity at the J-step (2 ms)
$F_I = F_{30ms}$	Fluorescence intensity at the I-step (30 ms)
$F_M = F_P$	Maximum recorded fluorescence, when all PSII RCs are closed
$S_M = A_M/(F_M - F_O)$, where A_M is the area above the OJIP curve between F_O and F_M and the F_M asymptote	Standardized area above the fluorescence curve between F_O and F_M is proportional to the pool size of the electron acceptors on the reducing side of

	Photosystem II
$V_J = (F_{2ms} – F_O)/(F_M – F_O)$	Relative variable fluorescence at J-step (2 ms)
$\Delta V_G/\Delta t_0$	Slope of prompt fluorescence curve rise, between 50 and 100 µs
$M_0 = 4\ (F_{300\mu s} – F_O)/(F_M – F_O)$	Approximated initial slope of the fluorescent transient. This parameter is related to rate of closure of reaction centers
**Specific energy fluxes expressed per active PSII reaction center (RC)**	
$ABS/RC = M_0 \times (1/V_J) \times [1 – (F_O/F_M)]$	Apparent antenna size of active PSII RC
$TR_0/RC = M_0 \times (1/V_J)$	Trapping flux leading to Q_A reduction per RC
$ET_0/RC = M_0 * (1/V_J) * \psi_0$,<br>where $\psi_0 = (1 – V_J)$	Electron transport flux per reaction center (RC) at t=0
$RE_0/RC = M_0(1/V_J)(1 – V_J)$	Quantum yield of electron transport from Q_A^- to the PSI end electron acceptors
$DI_0/RC = (ABS/RC) – (TR_0/RC)$	Dissipated energy flux per reaction center (RC) at t=0
RC/CS_0	Number of active PSII RCs per illuminated cross-section (CS) at initial moment of illumination (at t=0)
$N = (S_M/S_S)$, where $S_S = V_J/M_0$	Number indicating how many times Q_A is reduced while fluorescence reaches its maximal value (number of Q_A redox turnovers until F_M is reached); S_S – normalized curve above O-J curve.
**Quantum yields and probabilities**	
$\varphi_{Po} \equiv TR_0/ABS = [1 – F_O/F_M)] = F_V/F_M$	Maximum quantum yield of primary PSII photochemistry
$\varphi_{Eo} = (1 – F_J/F_M)(1 – V_J)$	Quantum yield for electron transport from Q_A^- to plastoquinone
$\varphi_{Ro} = (1 – F_I/F_M)(1 – V_J)$	Quantum yield for reduction of end electron acceptors at the PSI acceptor side (RE)

$\psi_0 = ET_0/TR_0 = (1 - V_J)$	probability (at t = 0) that a trapped exciton moves an electron into the electron transport chain beyond Q_A^- to plastoquinone
$\delta_0 = TR_0/ET_0/ = (1 - V_I)$	probability (at t = 0) of electron transport from reduced plastoquinone to PSI end electron acceptors
γ_{RC}	Probability, that PSII chlorophyll molecule function as RC
**Performance indexes and driving forces**	
$PI_{ABS}= \gamma_{RC/}(1 - \gamma_{RC}) \times\varphi_{Po}/(1 - \varphi_{Po}) \times\psi_o/(1 -\psi_o)$	Performance index of electron flux from PSII based to intersystem acceptors
$PI_{total}= PI_{ABS}\times \delta_{Ro}/(1 -\delta_{Ro})$,<br>where $\delta_{Ro}= (1 - V_J)/(1 - V_I)$	Performance index of electron flux to the final PSI electron acceptors
